# Mammalian *NPC1 *genes may undergo positive selection and human polymorphisms associate with type 2 diabetes

**DOI:** 10.1186/1741-7015-10-140

**Published:** 2012-11-15

**Authors:** Nasser M Al-Daghri, Rachele Cagliani, Diego Forni, Majed S Alokail, Uberto Pozzoli, Khalid M Alkharfy, Shaun Sabico, Mario Clerici, Manuela Sironi

**Affiliations:** 1 Biomarkers Research Program, Biochemistry Department, College of Science, King Saud University, King Abdullah road, Riyadh 11451, Kingdom of Saudi Arabia; 2 Prince Mutaib Chair for Biomarkers of Osteoporosis Research, King Saud University, King Abdullah road, Riyadh 11451, Kingdom of Saudi Arabia; 3 Center of Excellence in Biotechnology, King Saud University, King Abdullah road, Riyadh 11451, Kingdom of Saudi Arabia; 4 Bioinformatics, Scientific Institute IRCCS E.MEDEA, Via Don L. Monza 20, Bosisio Parini 23842, Italy; 5 Clinical Pharmacy Department, College of Pharmacy, King Saud University, King Abdullah road, Riyadh 11451, Kingdom of Saudi Arabia; 6 Department of Molecular Medicine, Don Gnocchi Foundation IRCCS, ONLUS, Piazetta Morandi, Milano 20100, Italy; 7 Department of Pathophysiology and Transplantation, Milano University Medical School, Via Fratelli Cervi, Milano 20090, Italy

**Keywords:** NPC1, filovirus, natural selection, type 2 diabetes

## Abstract

**Background:**

The *NPC1 *gene encodes a protein involved in intracellular lipid trafficking; its second endosomal loop (loop 2) is a receptor for filoviruses. A polymorphism (His215Arg) in *NPC1 *was associated with obesity in Europeans. Adaptations to diet and pathogens represented powerful selective forces; thus, we analyzed the evolutionary history of the gene and exploited this information for the identification of variants/residues of functional importance in human disease.

**Methods:**

We performed phylogenetic analysis, population genetic tests, and genotype-phenotype analysis in a population from Saudi Arabia.

**Results:**

Maximum-likelihood ratio tests indicated the action of positive selection on loop 2 and identified three residues as selection targets; these were confirmed by an independent random effects likelihood (REL) analysis. No selection signature was detected in present-day human populations, but analysis of nonsynonymous polymorphisms showed that a variant (Ile642Met, rs1788799) in the sterol sensing domain affects a highly conserved position. This variant and the previously described His215Arg polymorphism were tested for association with obesity and type 2 diabetes (T2D) in a cohort from Saudi Arabia. Whereas no association with obesity was detected, 642Met allele was found to predispose to T2D. A significant interaction was noted with sex (*P *= 0.041), and stratification on the basis of gender indicated that the association is driven by men (*P *= 0.0021, OR = 1.5). Notably, two *NPC1 *haplotypes were also associated with T2D in men (rs1805081-rs1788799, His-Met: *P *= 0.0012, OR = 1.54; His-Ile: *P *= 0.0004, OR = 0.63).

**Conclusions:**

Our data indicate a sex-specific effect of *NPC1 *variants on T2D risk and describe putative binding sites for filoviruses entry.

## Background

**Figure 1:**
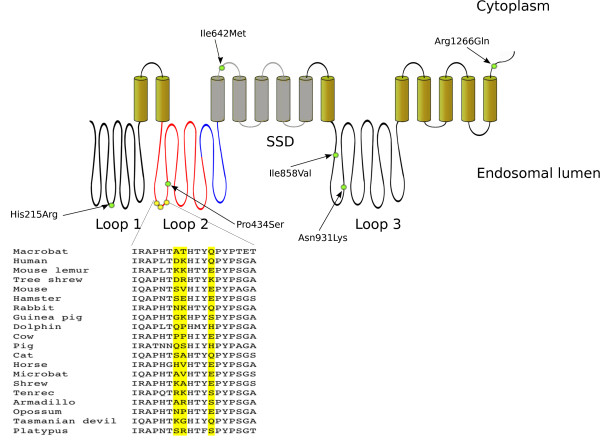
**Schematic representation of the NPC1 protein (not to scale)**. Cylinders represent the transmembrane regions; the SSD domain is depicted in grey. Luminal loop 2 is represented in blue and red to account for the recombination breakpoint. The position of the three positively selected sites in loop 2 is shown (yellow circles) with the alignment of a few representative mammalian species. The position of nonsynonymous polymorphisms with a minor allele frequency higher than 1% is represented by the green circles. SSD, sterol sensing domain

The *NPC1 *gene encodes a large multi-domain protein involved in the intracellular trafficking of sterols. Mutations in the gene are responsible for a rare and fatal lipid storage disorder, Niemann-Pick disease type C. The product of *NPC1 *resides in the limiting membrane of late endosomes and lysosomes where it facilitates lipid transport to various cellular compartments (reviewed in [1]). The protein displays 13 transmembrane domains, and three large loops are present in the lumen of the endosome (Figure 1) [2]. Interaction with lipid substrates is mediated by the most N-terminal luminal loop (loop 1) and by a sterol-sensing domain (SSD) which comprises five central transmembrane regions [2] (Figure 1). Recent works showed that the subcellular localization of NPC1 has been exploited by viruses of the *Filoviridae *family for host invasion [3-5]. Thus, viruses such as Ebola and Marburg require NPC1 protein expression for productive infection and the second luminal domain of NPC1 binds directly and specifically to the GP1 viral glycoprotein [3]. Consistently, primary fibroblasts from human Niemann-Pick type C1 disease patients are resistant to infection by filoviruses [4].

Mice lacking *Npc1 *function display a phenotype recapitulating Niemann-Pick disease type C [6], whereas haploinsufficiency for the gene results in weight gain and insulin resistance [7,8]. In fact, *Npc1^+/- ^*mice display increased adiposity and adipocyte hypertrophy; these animals also show dyslipidemia and higher plasma glucose levels compared to their wild-type litter mates. In line with this evidence, a nonsynonymous polymorphism (rs1805081, His215Arg) in the human *NPC1 *gene has recently been associated with severe and early onset obesity in European populations [9]. A subsequent study confirmed the predisposing role of rs1805081 to obesity and increased body mass index (BMI) in Europeans, but found no association between the variant and type 2 diabetes (T2D) or fasting plasma lipid levels [10]. Conversely, the effect on obesity risk and higher BMI of the *NPC1 *SNP in Asian populations is still controversial [11,12]. The molecular mechanisms underlying the association between genetic variation in *NPC1 *and metabolic phenotypes remain to be clarified. However, analysis of *Npc1 *mutant mice revealed that these animals are characterized by increased liver accumulation of triacylglycerol [7], higher hepatic expression of caveolin-1 [13], a protein involved in liver lipid metabolism [14], and of sterol regulatory element-binding proteins (SREBPs) [15]. These observations suggest that mutations or polymorphisms in *NPC1 *result in alteration of hepatic lipid homeostasis eventually leading to weight gain and insulin resistance.

Adaptations to diet and to pathogen exposure are thought to have represented a powerful driving force throughout the evolutionary history of mammals [16]. Thus, we performed a phylogenetic analysis of *NPC1 *genes in mammals and a population genetics study of diversity in human populations. We identified three residues that have been targets of positive selection, possibly mediated by filovirus-exerted selective pressure. No selection signature was detected in present-day human populations, but analysis of nonsynonymous polymorphisms identified a variant (Ile642Met) in the SSD domain that affects a highly conserved position. This variant and *NPC1 *haplotypes were found to modulate the risk of T2D (but not BMI or obesity) in a population from Saudi Arabia.

## Methods

### Evolutionary analysis

Most mammalian *NPC1 *sequences were retrieved from the Ensembl website [17]. The sequence of baboon was obtained though blast search in the National Center for Biotechnology Information (NCBI) Trace Archive against *Papio hamadryas *whole genome sequence. *NPC1 *coding sequences for *Cricetulus griseus *and *Mustela putorius *(C-terminal portion only) were retrieved from the NCBI nucleotide database (NM_001246687.1 and JP014452, respectively).

DNA alignment was performed using the The RevTrans 2.0 utility [18], which uses the peptide sequence alignment [see Additional file 1, Figure S1] as a scaffold for constructing the corresponding DNA multiple alignment. This latter was checked and edited by hand to remove alignment uncertainties. The alignment was used for Genetic Algorithm Recombination Detection (GARD) [19] analysis through the DataMonkey [20]. Similarly, the evolutionary selection distance (ESD), random effects likelihood (REL) and branch-site REL analyses were performed using DataMonkey [20]. For phylogenetic analysis by maximum likelihood (PAML) analyses we used multiple alignments of *NPC1 *sub-regions and trees generated by maximum-likelihood using the program DnaML (PHYLIP Package). To detect selection, Nssite models that allow (M8) or disallow (M7 and M8a) a class of codons to evolve with dN/dS >1 were fitted to the data using both the F61 (Table 1) and the F3X4 [see Additional file 1, Table S1] codon frequency models. Sites under selection for the M8 model were identified using Bayes empirical Bayes (BEB) analysis using a significance cutoff of 0.90 [21,22].

**Table 1:** Likelihood ratio test statistics for models of variable selective pressure among sites (F61 model of codon frequency).

Region/selection model(number of codons)	**d.f**.	-2ΔLnL	*P *value	% of sites(average dN/dS)	Selected sites
**Loop1 (269)**					
M7 versus M8	2	10.04	0.0066	1.3% (1.37)	182 (0.99, n.s.)
M8a versus M8	1	4.87	0.027	-	-
					
**Loop 2 N-term (251)**					
M7 versus M8	2	25.86	<0.0001	1.3% (1.78)	416 (0.99, 0.99),417 (0.98, 1), 421 (0.91, 0.99)
M8a versus M8	1	10.98	0.0009	-	
					
**Loop 2 C-term (81)**					
M7 versus M8	2	<0.01	>0.99	-	-
M8a versus M8	1	1.96	0.16		-
					
**SSD (164)**					
M7 versus M8	2	5.70	0.058	-	-
M8a versus M8	1	0.02	>0.88	-	-
					
**Loop 3 (248)**					
M7 versus M8	2	0.004	>0.99	-	-
M8a versus M8	1	1.52	0.21	-	-

M7 is a null model that assumes that 0<ω<1 is beta distributed among sites; M8a assumes 0<ω≤1; these two models are compared to M8, which includes an extra category of sites with ω>1 (positive selection). d.f., degree of freedom; 2ΔLnL, twice the difference of the natural logs of the maximum likelihood of the models being compared; *P *value, *P *value of rejecting the neutral models (M7 or M8a) in favor of the positive selection model (M8); % of sites (average dN/dS), estimated percentage of sites evolving under positive selection by M8 (dN/dS for these codons); selected sites, the position refers to the entire *NPC1 *human sequence, *P *values obtained from BEB and REL analyses are shown. BEB, Bayes empirical Bayes; REL, random effects likelihood; SSD, sterol sensing domain.

### Population genetic analyses

Data from the Pilot 1 phase of the 1000 Genomes Project were retrieved online [23]. Low-coverage SNP genotypes were organized in a MySQL database. A set of programs was developed to retrieve genotypes from the database and to analyze them according to selected regions/populations. These programs were developed in C++ using the GeCo++ [24] and the *libsequence *[25] libraries. Genotype information was obtained for *NPC1 *and for 2,000 randomly selected RefSeq genes.

Sliding window analysis was performed on overlapping 5 kb windows moving with a step of 500 bp. For each window we calculated θ_W_, π, and F_ST _and these values were used to obtain the empirical distributions and to calculate percentiles. Values for the integrated haplotype_score (iHS) for HapMap Phase II SNPs were derived from a previous work [26].

### Patients and controls

All subjects recruited in the study are part of the Biomarker Screening in Riyadh Project (RIYADH COHORT), a capital-wide epidemiologic study that has so far enrolled more than 17,000 Saudis from different Primary Health Care Centers. Demographic and medical information is recorded for all individuals participating in the program. DNA samples have been collected from more than 1,600 of these individuals. These individuals were selected to represent case-control cohorts for T2D. Subjects with medical complications (coronary artery disease, nephropathy, and end stage renal disease or liver disease) were excluded and a similar percentage of men and women were enrolled among T2D patients and controls. After discarding samples with poor DNA quality, 1,468 subjects were included in the study (644 T2D, 52% women; 824 controls, 54% women). Diagnosis of T2D was based on the World Health Organization proposed cut-off (fasting plasma glucose > or = 7.0 mmol/L or 126 mg/dl) as previously described [27].Written consent was obtained from all participants, and ethical approval was granted by the Ethics Committee of the College of Science Research Center, King Saud University, Riyadh, Kingdom of Saudi Arabia (KSA).

### Anthropometry and DNA extraction

After an overnight fast, subjects underwent anthropometry and blood withdrawal. Anthropometry included measurement of height (to the nearest 0.5 cm) and weight (to the nearest 0.1 kg); BMI was calculated as kg/m^2^. According to the World Health Organization (WHO) criteria, individuals were classified as obese if their BMI was > 30 kg/m^2^. Whole blood was collected in ethylenediaminetetraacetic acid (EDTA)-containing tubes; genomic DNA was isolated using the blood genomic prep minispin kit (GE Healthcare, Milano, Italy). Genotyping and statistical analysisThe two *NPC1 *SNPs were genotyped by allelic discrimination real-time PCR, using predesigned TaqMan probe assays (Applied Biosystems, Foster City, CA, USA). Reactions were performed using TaqMan Genotyping Master Mix in an ABI 9700 analyzer (Applied Biosystems). Genotyping rate was >0.97 for both variants. In the text and tables, the allelic status of the two variants is shown with reference to the transcript orientation with the ancestral allele reported first. Genetic association was investigated by multiple linear or logistic regression (as appropriate) using genotypes/haplotypes as the independent predictor variables with sex and age as covariates; BMI was added as a covariate when addressing the association between T2D and *NPC1 *variants; T2D was accounted for when addressing the effect of SNPs/haplotypes on obesity and BMI. Before carrying out parametric statistical procedures, total cholesterol and triglyceride levels were logarithmically transformed to ensure a more normal distribution. Analyses were performed using PLINK [28].

## Results

### Evolutionary analysis of *NPC1 *mammalian genes

**Figure 2:**
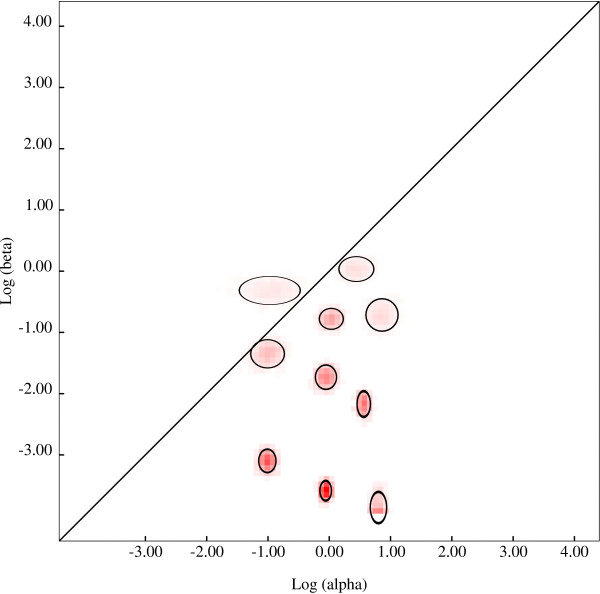
**Evolutionary fingerprint of *NPC1***. The estimate of the distribution of synonymous and nonsynonymous substitution rates is plotted on a log-log scale. The ellipses reflect a Gaussian-approximated variance in each individual rate estimate, and colored pixels show the density of the posterior sample of the distribution for a given rate. The diagonal line represents the neutral evolution expectation (ω = 1), points above the line correspond to positive selection (ω >1), and points below the line to purifying selection (ω <1)

To analyze the evolutionary history of *NPC1 *in mammals we retrieved coding sequence information for 41 species from public databases (see methods). Alignment of these sequences revealed that *NPC1 *evolved under purifying selection, as the average non-synonymous substitution rate (dN) was generally much lower than the rate for synonymous substitutions (dS) (average dN/dS = 0.12). Nonetheless, natural selection might act on a few sites within a gene that is otherwise strongly constrained. Before testing this possibility, we screened the *NPC1 *alignment for evidence of recombination using a recently developed algorithm (GARD) [19]; this analysis uncovered the presence of one single recombination breakpoint at nucleotide position 1619 (ΔAIC_c _= 53.7), falling within luminal loop 2 (Figure 1). After taking this information into account, we analyzed the evolutionary fingerprint of *NPC1 *by applying the ESD method [29], which uses the site-by-site probability distribution of synonymous and nonsynonymous substitution rates to partition sites into selective classes. ESD estimated 10 substitution rate classes (Figure 2), one of which showing dN/dS (ω) >1, indicative of positive selection. Specifically, the estimated average ω for this class was 1.98 with an estimated percentage of sites of 2% (95% IC: 0.1 to 0.3). We next applied maximum-likelihood analyses implemented in the PAML package [30,31] to single *NPC1 *domains. Specifically, we separately analyzed luminal loops 1 and 3, as well as the SSD domain; luminal loop 2 was divided into two halves to account for the recombination breakpoint. Results indicated that a model allowing sites to evolve with ω >1 (M8) had significantly better fit to the data than models assuming no positive selection (M7 and M8a) for the N-terminal portion of loop 2 (Table 1, and Additional file 1, Table S1). Some evidence of positive selection was also evident for loop 1. No selection signature was detected for the remaining *NPC1 *regions. Three sites in the N-terminal portion of loop 2 were found to have a high posterior probability of being under positive selection according to BEB analysis (*P *>0.90) (Table 1 Figure 1) [21,22]. These three sites were confirmed by an independent REL analysis that allows variation of dS among sites [32] (Table 1). BEB analysis also identified one site in luminal loop 1, which was not confirmed by REL analysis. Finally, we verified whether any lineage shows evidence of episodic positive selection by applying a branch-site REL analysis [33]. Results indicated that a proportion of sites has evolved under episodic diversifying selection in the gorilla and baboon lineages, although the proportion of sites evolving with ω >1 was very low (about 1%) in both lineages. Thus, the branch-site REL test should be interpreted with caution, as sequencing errors in the reference sequences of these two primates might be partially responsible for these results [see Additional file 1, Figure S2].

### Population genetics in humans

The human *NPC1 *gene spans about 55 kb on chromosome 18. To gain insight into its evolutionary history in human populations, we exploited sequencing data from the 1000 Genomes Pilot Project [34], which generated low-coverage whole genome sequencing data of 179 individuals with different ancestry (Yoruba from Nigeria, Europeans and Asians). Nucleotide diversity for the entire *NPC1 *gene region was calculated using θ_W, _an estimate of the expected per site heterozygosity [35] and π, the average number of pair-wise sequence nucleotide differences between haplotypes [36]. As a comparison, the same indexes were obtained for 2,000 randomly selected human genes. Both θ_W _and π for *NPC1 *ranged from the 29th to the 40th percentiles in the distribution of values calculated for the 2,000 reference genes in the three populations (not shown). In order to address the possibility of local selection affecting *NPC1 *sub-regions, we performed a sliding window analysis of θ_W_, π, and Yoruba/European/Asian population genetic differentiation (F_ST_) [37] along the gene. Again, we applied the same procedure to 2,000 randomly selected human genes, allowing calculation of the 2.5th and 97.5th percentiles to be used as reference cutoffs. No region in NPC1 displayed nucleotide diversity outside the calculated cutoffs [see Additional file 1, Figure S3]. As for F_ST_, a peak was evident in the middle of the gene, but it did not exceed the 97.5th percentile [see Additional file 1, Figure S4]. Analysis of iHS [26] for variants within the peak revealed no absolute value higher than 2 (data not shown). Overall, these analyses suggest that NPC1 is neutrally evolving in humans or that selection signatures are too weak to be detected using these approaches.

### Association of *NPC1 *SNPs with obesity and T2D

To shed light on the distribution of polymorphisms segregating in *NPC1 *we again exploited the 1000 Genomes Project data [34] by selecting nonsynonymous variants that have been detected in the gene with a minor allele frequency higher than 1%. Six variants were identified; only two of them were located in domains possibly affecting sterol homeostasis: rs1805081 (His215Arg), located in loop 1 and previously associated with obesity in Europeans [9], and rs1788799 (Ile642Met), located in the SSD (Figure 1). Analysis of the mammalian *NPC1 *alignment indicated that codon 215 is relatively variable, whereas position 642 is conserved (Ile) in all species [see Additional file 1, Figure S1]. We analyzed the role of these two SNPs in predisposing to obesity and weight gain by recruiting a population consisting of 1,468 subjects (820 obese individuals and 648 non-obese controls) from Saudi Arabia (Table 2). The two polymorphisms displayed limited linkage disequilibrium (LD) in our study population (D' = 0.93, r^2 ^= 0.080) and both complied with Hardy-Weinberg equilibrium. Minor allele frequencies for rs1788799 (G, 642Met) and rs1805081 (G, 215Arg) in this cohort amounted to 0.41 and 0.12, respectively. Association of these SNPs with obesity was assessed by fitting a logistic regression model using age, sex, and absence/presence of T2D as covariates. Results indicated that neither SNP associates with obesity (Table 3). Similarly, no association between *NPC1 *variants and BMI was detected (Table 3).

**Table 2:** Characteristics of the Saudi cohort.

	Obese		Non-obese	
	
	T2D	No T2D	T2D	No T2D
Sample size	441	379	203	445
Females (%)	261 (59)	233 (61)	73 (36)	216 (48.5)
Males (%)	180 (41)	145 (39)	130 (64)	229 (51.5)
Age ± s.d. (years)	53.07 ± 10.61	42.79 ± 10.15	55.54 ± 16.19	38.56 ± 13.90
BMI ± s.d., kg/m^2^	35.62 ± 6.83	34.67 ± 3.92	24.25 ± 3.31	22.74 ± 3.3

T2D, type 2 diabetes.

**Table 3:** Association analysis of ***NPC1 *polymorphisms with obesity, BMI, and T2D**.

Trait	Minor allele	Whole sample		Males		Females	
	
		*P *value^a^	OR (95% CI)^b^	*P *value^a^	OR (95% CI)^b^	*P *value^a^	OR (95% CI)^b^
**Obesity**							
rs1788799 (C/G)	G (Met)	>0.5	1.05 (0.87 to 1.18)	>0.5	1.02 (0.81 to 1.27)	>0.5	1.02 (0.83 to 1.27)
rs1805081 (A/G)	G (Arg)	>0.5	0.83 (0.78 to 1.25)	>0.5	1.06 (0.76 to 1.47)	>0.5	0.93 (0.67 to 1.30
							
**BMI**		***P *value^a^**	**BETA^c^**	***P *value^a^**	**BETA^c^**	***P *value^a^**	**BETA^c^**
rs1788799 (C/G)	G (Met)	0.462	0.328	>0.5	0.490	>0.5	0.231
rs1805081 (A/G)	G (Arg)	>0.5	-0.411	>0.5	-0.343	>0.5	-0.450
							
**T2D**		***P *value^a^**	**OR (95% CI)^b^**	***P *value^a^**	**OR (95% CI)^b^**	***P *value^a^**	**OR (95% CI)^b^**
rs1788799 (C/G)	G (Met)	**0.027**	1.24 (1.05 to 1.48)	**0.004**	1.50 (1.15 to 1.95)	>0.5	1.07 (0.85 to 1.36)
rs1805081 (A/G)	G (Arg)	>0.5	1.06 (0.82 to 1.39)	>0.5	1.11 (0.76 to .60)	>0.5	1.04 (0.71 to 1.52)

^a^*P *values were calculated using logistic or linear regression (as appropriate) with an additive model; Bonferroni-corrected *P *values are reported; nominally significant *P *values are in bold; ^b^odds ratio and 95% confidence intervals refer to the minor allele. ^c^regression coefficient. BMI, body mass index; OR, odds ratio; T2D, type 2 diabetes.

We next evaluated the role of rs1805081 and rs1788799 in predisposing to T2D; to this aim all subjects were analyzed by fitting a logistic regression using age, sex, and BMI as covariates. No effect of rs1805081 on T2D susceptibility was observed; conversely, a significant association between rs1788799 and T2D was detected (for the minor allele 642Met, *P *= 0.0137, odds ratio (OR) = 1.24) (Table 3). A significant interaction was also noted between allelic status at this variant and sex (*P_interaction _*= 0.041); stratification of the population on the basis of gender indicated that the association between rs1788799 and T2D is driven by male subjects (Table 3). Thus, we next analyzed the effect of *NPC1 *haplotypes on susceptibility to diabetes. After correcting for age, sex and BMI, two haplotypes were found to be associated with T2D with an opposite effect. Specifically, AC and AG (rs1805081-rs1788799, 215His-642Ile and 215His-642Met) haplotypes were observed to protect and predispose to the disease, respectively (Table 4). Again, the association could only be detected in men and occurred in both obese and non-obese individuals (Table 4).

**Table 4:** Association analysis of *NPC1 *haplotypes with T2D.

Trait	Haplotype _(rs1805081 -rs1788799, residues)_	Whole sample		Males		Females	
	
		Freq.^a^	*P *value (OR)	Freq.^a^	*P *value (OR)	Freq.^a^	*P *value (OR)
T2D (all)							
	GC (Arg-Ile)	0.12	0.451 (1.11)	0.12	0.495 (1.14)	0.12	0.653 (1.09)
	AG (His-Met)	0.41	**0.009 **(1.26)	0.40	**0.0012 (1.54)**	0.41	0.545 (1.08)
	AC (His-Ile)	0.47	**0.003 **(0.77)	0.48	**0.0004 (0.63)**	0.47	0.425 (0.91)
							
T2D (obese)							
	GC (Arg-Ile)	0.12	0.577 (1.10)	0.12	0.498 (1.20)	0.12	0.780 (1.07)
	AG (His-Met)	0.42	0.230 (1.14)	0.42	**0.013 **(1.44)	0.41	0.745 (0.95)
	AC (His-Ile)	0.46	0.142 (0.85)	0.46	**0.004 **(0.60)	0.47	0.813 (1.03)
							
T2D (non-obese)							
	GC (Arg-Ile)	0.11	0.77 (1.07)	0.11	0.957 (1.02)	0.12	0.666 (1.19)
	AG (His-Met)	0.40	**0.0125 **(1.46)	0.40	**0.033 **(1.52)	0.41	0.158 (1.40)
	AC (His-Ile)	0.49	**0.009 **(0.67)	0.49	**0.042 **(0.69)	0.47	0.088 (0.65)

^a^haplotype frequency. Significant *P *values are in bold. Freq., frequency; T2D, type 2 diabetes.

Finally, we evaluated the role of *NPC1 *haplotypes in modulating fasting plasma lipid levels. Circulating levels of total-, LDL- and HDL-cholesterol, as well as triglycerides were available for 1,443 individuals of the above described cohort. No effect of *NPC1 *haplotypes on total- and LDL-cholesterol was detected (Table 5). Conversely, different *NPC1 *haplotypes were associated, although weakly, with HDL-cholesterol and triglyceride levels both in men and women (Table 5).

**Table 5:** Association analysis of *NPC1 *haplotypes with lipid levels.

Trait	Haplotype _(rs1805081-rs1788799, residues)_	Whole sample		Males		Females	
	
		BETA^a^	*P *value	BETA^a^	*P *value	BETA^a^	*P *value
HDL-cholesterol							
	GC (Arg-Ile)	-0.047	**0.026**	-0.070	**0.017**	-0.020	0.698
	AG (His-Met)	-0.013	0.32	0.004	0.803	-0.0278	**0.048**
	AC (His-Ile)	0.032	**0.015**	0.025	0.190	0.040	**0.023**
							
LDL-cholesterol							
	GC (Arg-Ile)	-0.068	0.650	-0.140	0.477	-0.034	0.877
	AG (His-Met)	0.149	0.124	-0.125	0.330	0.200	0.157
	AC (His-Ile)	-0.105	0.265	-0.701	0.583	-0.144	0.289
							
Total cholesterol							
	GC (Arg-Ile)	-0.011	0.408	0.003	0.872	-0.028	0.166
	AG (His-Met)	0.010	0.224	0.002	0.873	0.022	0.097
	AC (His-Ile)	-0.005	0.538	-0.003	0.794	-0.008	0.505
							
Triglycerides							
	GC (Arg-Ile)	0.045	0.101	0.104	**0.003**	-0.0313	0.466
	AG (His-Met)	0.024	0.173	-0.014	0.563	0.071	**0.009**
	AC (His-Ile)	-0.041	**0.018**	-0.031	0.181	-0.052	**0.046**

^a^regression coefficient. Significant *P *values are in bold.

## Discussion

During mammalian evolution genes involved in diet and immune response have been preferential targets of positive selection [16], highlighting the role of nutrient availability/preferences and pathogens as powerful selective forces. The protein product of *NPC1 *plays a central role in lipid metabolism, as it acts as a cholesterol transporter and its transcription is regulated by the SREBP pathway [1]. Conversely, the gene does not participate in immune response, but is exploited by members of the filovirus family as an intracellular receptor that mediates the late steps of viral invasion [3-5]. Evidence has indicated that genes directly involved in antiviral response or acting as viral receptors (for example, *HAVCR1, CD4*) display domains evolving under positive selection as the result of a genetic conflict with extant or extinct viral species [38-46]. Positive selection at these host genes may result from adaptation either to increase viral recognition and restriction efficiency or to avoid binding of specific viral components. Our evolutionary analysis in mammals indicated a predominant role of purifying selection in driving the evolution of *NPC1 *but also identified few positions that have been targeted by positive selection. Specifically, maximum-likelihood ratio tests indicated that three residues in the N-terminal portion of luminal loop 2 evolved under positive selection; these codons are located in close proximity to each other, and selection was confirmed by an independent REL analysis. PAML also identified one positively selected site in luminal loop 1, but this was not supported by REL, suggesting that it may represent a false positive, as the M8 model has been shown to be more prone than REL to false positive results when a relatively high number of sequences (species) is used for analysis [47]. These results suggest that the selective pressure responsible for positive selection in *NPC1 *stems from pathogens rather than from dietary changes. Indeed, a recent study has indicated that luminal loop 2 is necessary and sufficient to bind filovirus GP1 protein directly and to mediate productive infection [3]; the authors were able to map the GP1 residues involved in engaging loop 2 and determined that they are conserved among filoviruses [3]. This observation, together with evidence showing that NPC1 is required for infection of both human and rodent cells by distantly related viral species, strongly suggests that the cholesterol transporter is a necessary factor for most members of the *Filoviridae *family [3-5]. These pathogens display a wide host range in mammals [48] and are thought to have affected vertebrates for millions of years, as testified by the detection of filovirus-derived elements in the genome of both eutherians and marsupials [49]. Thus, we suggest that the positively selected sites we identified in luminal loop 2 evolved in response to a host-filovirus arms race and might represent relevant residues in mediating GP1 binding.

Population genetic analysis of *NPC1 *in humans revealed no evident signature of natural selection in loop 2 or any other gene region, although we cannot exclude that weak or geographically-restricted selective events have acted on the gene. With respect to filovirus infection, this might not be surprising as the known human pathogens Ebola and Marburg viruses are highly virulent agents that rapidly kill infected individuals, a feature that possibly limits their spreading in human populations [50] and makes them unlikely candidates to play a role as selective agents. Genetic diversity in human *NPC1 *has nevertheless been recently associated with metabolic dysfunction, this association being based on the central role of the gene in lipid trafficking. Specifically, the His215Arg (rs1805081) variant in luminal loop 1, which is involved in cholesterol binding, was shown to associate with obesity in populations of European descent [9,10]. It has been proposed that alleles responsible for obesity and T2D might have evolved as 'thrifty' variants in ancient populations [51,52]. In line with this hypothesis, selection signatures have been detected for a few polymorphisms associated with these conditions [53,54], although this does not seem to be the case for *NPC1*. Nonetheless, inspection of nonsynonymous SNPs located in the gene revealed that, in addition to the above mentioned variant in loop 1, a polymorphism (Ile642Met, rs1788799) in the SSD domain segregates at relatively high frequency in human populations and affects an isoleucine residue which is conserved in all the mammals we analyzed.

We thus reasoned that this SNP might affect *NPC1 *function and modulate metabolic phenotypes. We tested this hypothesis in a large cohort of subjects from Saudi Arabia, a region where the prevalence of obesity and T2D is very high [55-57]. The previously described association between rs1805081 and obesity [9,10] was not replicated in the Saudi sample, although the relatively lower minor allele frequency (MAF) of the variant in this population (12%) compared to Europeans (ranging from 25% to 40%) might have limited our detection power. No effect on BMI or obesity was detected in the Saudi cohort for the Ile642Met variant either. Similarly, the role of the His215Arg variant in predisposing to obesity was not observed in a cohort of Chinese children [12], although a possible interaction between this (and other) variant and sedentary behavior has been described in a population of the same ethnicity [11]. Recently, a meta-analysis of rs1805081 on obesity risk in Europeans also revealed a weak effect of the polymorphism on body fat percentage, but not on BMI or on the odds of being obese [58]. One possibility to explain these contrasting results is that variants in *NPC1 *interact with environmental cues, as suggested by the Chinese study [11] and possibly with additional genetic factors. This seems to be the case for *Npc1^+/- ^*mice: these animals develop increased adiposity and metabolic disturbances but the phenotype depends on both fat intake and genetic background [7,59]. These animals also present with increased fasting plasma glucose levels, glucose intolerance, and insulin resistance, indicating a T2D phenotype [7,59]. Somehow in contrast with these results, a recent study indicated that heterozygosity for a hypomorphic *Npc1 *mutation on the C57BL/6J 'metabolic syndrome' genetic background protects old male mice, but not females, from weight gain [60]. Overall, these observations suggest that *Npc1 *genetic variation interacts with diet, sex and with one or more gene(s) in modulating metabolic phenotypes.

A possible association between the two *NPC1 *variants and T2D was analyzed in the Saudi cohort. Overweight and obesity are strong risk factors for the development of T2D; genetic susceptibility is nevertheless believed to play a stronger role in non-obesity related T2D [61]. Thus, we verified the effect of rs1805081 and rs1788799 on diabetes susceptibility by taking BMI into account; a significant association was detected between rs1788799 and T2D, with a predisposing role for the derived 642Met allele.

Several metabolic traits are sexually dimorphic in humans and/or show sex-specific heritability linked to the autosomes [62]. Thus, it was suggested that variants with a sex-specific effect might be difficult to detect without separating the sexes or modeling for gender-based differences [62]. Testing for interaction with sex in our cohort indicated the presence of a significant effect; stratification of the population on the basis of gender revealed that the association is driven by male subjects. This was even more evident when haplotype analysis using the two coding variants was performed. Notably, two major haplotypes showed an opposite effect on T2D susceptibility in men only, and the effect was evident in both obese and non-obese individuals. An interaction between gender and genetic factors has been described for some other genes involved in T2D [63-66]; the reasons underlying these sex-specific events remain to be elucidated and might include a role for sex hormones, epistatic effects with X-linked variants, or differences in dietary habits and lifestyle between the sexes that, in turn, interact with the genetic status.

Further analyses on plasma lipid levels showed the presence of different associations with *NPC1 *haplotypes in men and women. Nonetheless, these effects were generally weak and should be interpreted with caution. The stronger effect was detected for triglyceride levels. Thus, in men a minor haplotype unrelated to T2D susceptibility was found to associate with higher levels, whereas in women the two major haplotypes that predispose or protect men from diabetes were found to be associated with higher and lower triglyceride levels, respectively.

## Conclusions

Data reported here indicate that *NPC1 *has evolved adaptively in mammals and that the underlying selective pressure might be virus-driven. No selection signature was detected in present-day human populations, but analysis of nonsynonymous polymorphisms showed that a variant (Ile642Met) in the SSD domain affects a highly conserved position. This variant and haplotypes comprising Ile642Met and the previously described His215Arg polymorphism were found to modulate the risk of T2D in a population from Saudi Arabia with a sex-specific effect. Analysis of additional cohorts will be instrumental for clarifying the role of the two *NPC1 *variants on plasma lipid levels and T2D susceptibility. Our results indicate that haplotype analysis (as opposed to single variant association) and modeling for sex-specific effects are strongly recommended when *NPC1 *genetic variability is analyzed.

## Abbreviations

BEB: Bayes empirical Bayes; BMI: body mass index; bp: base pair; ESD: evolutionary selection distance; GARD: Genetic Algorithm Recombination Detection; iHS: integrated haplotype score; NCBI: National Center for Biotechnology Information; OR: odds ratio; PAML: phylogenetic analysis by maximum likelihood; PCR: polymerase chain reaction; REL: random effects likelihood; SNP: single nucleotide polymorphism; SREBP: sterol regulatory element-binding proteins; SSD: sterol sensing domain; T2D: type 2 diabetes.

## Competing interests

The authors declare that they have no competing interests.

## Authors' contributions

MS, RC, DF and UP performed the experiments and analyzed the data. MC and MS designed the study and contributed to writing the manuscript. NMA contributed to the study design and to writing the manuscript. MSA, KMA and SS are responsible for following the cohorts of patients and collecting and cataloguing the samples. All authors read and approved the final manuscript.

## Pre-publication history

The pre-publication history for this paper can be accessed here:

http://www.biomedcentral.com/1741-7015/10/140/prepub

## Supplementary Material

Additional file 1 **Table S1: Likelihood ratio test statistics for models of variable selective pressure among sites**. The table reports results of the likelihood ratio tests (M7 versus M8 and M8a versus M8) using the F3X4 codon frequency model. **Figure S1: Multiple protein alignment of *NPC1 *mammalian genes**. The figure shows the *NPC1 *multiple species alignment (41 species, Clustal format); positively selected sites and human nonsynonymous polymorphisms are highlighted. **Figure S2: Branch-site random effects likelihood (branch-site REL) analysis of *NPC1 *genes**. The figure shows a branch-site REL analysis of *NPC1 *with the width and color of each branch indicating the strength of selection. **Figure S3: Sliding-window analysis of nucleotide diversity along *NPC1 *using the 1000 Genomes Project data**. The figure shows θW and π calculated for Yoruba, Europeans and Asians in sliding windows of 5 kb moving along the *NPC1 *gene region. **Figure S4**: **Sliding-window analysis of FST along *NPC1 *using the 1000 Genomes Project data**. The figure shows F_ST _(YRI/CEU/CHB-JPT) calculated in 5 kb windows moving along the *NPC1 *gene region. Click here for file
