# Supplementary material for: Mammalian NPC1 genes may undergo positive selection and human polymorphisms associate with type 2 diabetes
Source: BMC Med. 2012 Nov 15;10:140. doi: 10.1186/1741-7015-10-140 (PMC3520752; doi:10.1186/1741-7015-10-140)

Mammalian *NPC1* genes may undergo positive selection and human polymorphisms associate with type 2 diabetes

Nasser M. Al-Daghri[1,2,3] , Rachele Cagliani[4], Diego Forni[4], Majed S. Alokail[1,2,3], Uberto Pozzoli[4], Khalid M. Alkharfy[1,2,3,5], Shaun Sabico[1,2,3], Mario Clerici[6,7]*, Manuela Sironi[4]*

[1]Biomarker research program, Biochemistry Department, College of Science, King Saud University, Riyadh 11451, Kingdom of Saudi Arabia (KSA); [2]Prince Mutaib for biomarkers of osteoporosis, King Saud University, Riyadh 11451, KSA; [3]Center Of Excellence in Biotechnology, King Saud University, Riyadh 11451, KSA; [4]Scientific Institute IRCCS E.MEDEA, Bosisio Parini, Italy, [5]Clinical Pharmacy Department, College of Pharmacy, King Saud University, Riyadh 11451, KSA, [6]Don Gnocchi Foundation, ONLUS, Milano; [7]Milano University Medical School, Milano 20090, Italy

* these authors equally contributed to this work

Address for Correspondence: Nasser M. Al-Daghri, PhD, Biomarkers Research Program, Biochemistry Department, College of Science, King Saud University. PO Box, 2455, Riyadh, 11451 Kingdom of Saudi Arabia. Tel No: 0096614675939; Fax No: 0096614675931;

E-Mail: aldaghri2011@gmail.com

Supplemental Tables and Figures

Supplemental Table 1. Likelihood ratio test statistics for models of variable selective pressure among sites (F3X4 codon frequency model)

Region/selection model	d.f.	2ΔLnL	P value	% of sites (average dN/dS)	Selected sites (position, BEB p value, REL p value)
**Loop 1**					
M7 vs M8	2	8.25	0.016	0.7% (1.5)	182 (0.99, n.s)
M8a vs M8	1	4	0.045	-	-
**Loop 2 N-term**					
M7 vs M8	2	34.3	<0.0001	3.3% (1.325)	416 (0.98, 0.99), 417 (0.99, 1), 421 (0.91, 0.99)
M8a vs M8	1	11.08	0.0009	-	-
**Loop 2 C-term**					
M7 vs M8	2	0.04	0.98		
M8a vs M8	1	2.84	0.092		
**SSD**					
M7 vs M8	2	5.16	0.075	-	-
M8a vs M8	1	2	0.16	-	-
**Loop 3**					
M7 vs M8	2	7.36	0.025	-	-
M8a vs M8	1	3.62	0.057	-	-

Note: M7 is a null model that assumes that $0<\omega<1$ is beta distributed among sites; M8a assumes $0<\omega\leq1$; these two models are compared to M8, which includes an extra category of sites with $\omega>1$ (positive selection). The F3X4 codon frequency model was used. D.f.: degree of freedom; 2ΔLnL: twice the difference of the natural logs of the maximum likelihood of the models being compared; p value: p value of rejecting the neutral models (M7 or M8a) in favor of the positive selection model (M8); % of sites (average dN/dS): estimated percentage of sites evolving under positive selection by M8 (dN/dS for these codons); selected sites: the position refers to the entire NPC1 human sequence, p values obtained from BEB and REL analyses are shown.

Supplemental Figure 1. Multiple protein alignment of *NPC1* mammalian genes. Sites subject to positive selection are highlighted in yellow; the position of human nonsynonymous polymorphisms is shown in green.

```
Tenrec              MRARSLALG-LLLLLCPAQVFSQSCVWYGECGIATGDKRYNCEYSGPAKPLPKDGYDLVQ
Macrobat            MGARGPALGLLLLLLLCPAQVFSHSCVWYGECGIASGDKRYNCKYSGPPKLLPKDGYDLVQ
Hyrax               ----MSALCILLLLLLCLGQVFSQSCVWYGECGIAAGDKRYNCRYSGPPKPLPKDGYDLVQ
Tree shrew          -------------------VFSQSCVWYGECGIATGDKRYNCKYSGPPKPLPKDGYDLVQ
Armadillo           --MRGRDLAPLLLLLCPAQVFSQSCVWYGECGIATGDKRYNCKYSGPPVPLPDAGYDLVQ
Kangaroo rat        MKARGPALGLLLLLLLCPAQXXXXXXXXXXXXXXXXXXXXXXXXXXXXXXXXXXXXXXXXX
Shrew               MSARGRTVG-LLLLLSVAQVCAQSCVWYGECGIASGDKRYNCEYSGPPKPLPKDGYDLVQ
Dolphin             MSACGPALGLLLLLLLCPAQVFSQSCVWYGECGIASGDKRYNCRYSGLPKPLPEDGYDLVQ
Cow                 MSVRGPAFGLLLLLFCPAQVFSQSCIWYGECGIASGDKRYNCRYSGPPEPLPQDGYDLVQ
Pig                 MSARGPAFGLLLLLLCPVQVFSQSCVWYGECGIAYGDKRYNCRYSGPPKPLPEDGYDLVQ
Weasel              XXXXXXXXXXXXXXXXXXXXXXXXXXXXXXXXXXXXXXXXXXXXXXXXXXXXXXXXXXXXX
Panda               MTARGPALGLLLLLLLCPAQVFAQACVWYGECGIASGDKRYNCQYSGPPKPLPKDGYDLMQ
Dog                 --------------------FAQSCVWYGECGIASGDKRYNCQYSGPPKPLPKDGYDLMQ
Cat                 MTARGPALGLLLLLLLCPAQVLAQSCIWYGECGIASGDKRYNCKYSGPPKPLPKDGYDLVQ
Microbat            MSARGLALG-LLLLLCPAQVFPQSCVWYGECGIASGDKRYNCEYSGPPKALPKDGYDLVQ
Horse               MSAPGPALDLLLLLLCAAQVFSQSCVWYGECGIAFGDKRYNCEYSGPPKPLPKDGYDLLQ
Mouse lemur         MGARGPALG-LLLLLCPAQVFLQSCVWYGECGIASGDKRYNCKYSGPPKPLPKDGYDLVQ
Pika                -------------------VIPQSCVWYG-----------------------------VR
Rabbit              VSARGRLLGLLLLLLCPAQVLPQSCVWYGECGIASGDKRYNCQYSGPPKPLPKDGYDLIQ
Orangutan           MTARGLALGLLLLLLCPAQVFSQSCVWYGECGIAYGDKRYNCEYSGPPKPLPKDGYDLVQ
Human               MTARGLALGLLLLLLCPAQVFSQSCVWYGECGIAYGDKRYNCEYSGPPKPLPKDGYDLVQ
Chimpanzee          MIARGLALGLLLLLLCPAQVFSQSCVWYGECGIAYGDKRYNCEYSGPPKPLPKDGYDLVQ
Gorilla             MTARGLALGLLLLLLCPAQVFSQSCVWYGECGIAYGDKRYNCEYSGPPKPLPKDGYDLVQ
Marmoset            -------------------VFSQSCVWYGECGMAYGDKRYNCEYSGPPKPLPKDGYDLVQ
Macaque             MSARGLALGLLLLLLCLAQVFSQSCVWYGECGIAYGDKRYNCEYFGPPKPLPKDGYDLVQ
Gibbon              XXXXXXXXXXXXXXXXXXXXXXXXXXXXXXXXXXXXXXXXXXXXXXXXXXXXXXXXXXXXX
Baboon              MSARGLALGLLLLLLCPAQVFSQSCVWYGECGIAYGDKRYNCEYFGPPKPLPKDGYDLVQ
Bushbaby            MSALGPALRILLLLLCPAQVFSQSCVWYGECGIASGDKRYNCKYSGPPKPLPKDGYDLVQ
Tarsier             MSARGPALGLLLLLLWPAQVFSQSCVWYGECGIASGDKRYNCKYSGPPKPLPKDGYDLVQ
Mouse               MGAHHPALGLLLLLLCPAQVFSQSCVWYGECGIATGDKRYNCKYSGPPKPLPKDGYDLVQ
Rat                 MGAHHPALGLLLLLLCPAQVFSQSCVWYGECGVAFGDKRYNCEYSGPPKPLPKDGYDLVQ
Hamster             MSARHPALGLLLLLLCPAQVFSQSCVWYGECGIAFGDKKYNCKYSGPPKPLPKDGNDLLQ
Guinea pig          --------------------FSQSCIWFGECGIASGDKRYNCKYSGPPKPLPKDGYDLVQ
Opossum             -------------------VFSQSCVWYGECGMASEGNRYNCEYSGPPKPLPKDGYDLVQ
Tasmanian devil     ------------------------------------------------------------
Platypus            --------------------FSQSCIWYGECGIASGDKRYNCEYSGPPIPLPEDGYDLIQ
Elephant            MRARSPALSILLLLLCLAQVFSQSCVWYGECGIAAGDKRYNCKYSGPPKPLPKDGYDLVQ
Wallaby             -------------------VFSQSCVWYGECDIASEGKRYNCEYSGPPKPLPKDGYDLVQ
Sloth               XXXXXXXXXXXXXXXXXXXXXXXXXXXXXXXXXXXXXXXXXXXXXXXXXXXXXXXXXXXXX
Hedgehog            -------------------VFTQSCIWYGECGIASGDKRYNCKYSGPPKPLPKDGYDLVQ
Squirrel            -------------------VFLQSCVWYGECGIASGDKRYNCRYSGPPKPLPKDGYDLVQ


Tenrec              ELCPGFFFDSVNLCCDVQQLRTLKDNLQLPLQFLSRCPSCFYNLMNLFCELTCSPRQSEF
Macrobat            ELCPGFFSDNISLCCDVQQLQTLKDNLQLPLQFLSRCPSCFYNLLNMFCELTCSPRQSE-
Hyrax               ELCPGFFFDNVSLCCDVQQLRTLKDNLQLPLQFLSRCPSCFYNLMNLFCELTCSPRQSWF
Tree shrew          ELCPGFFFDNVSLCCDVQQLQTLKDNLQLPLQFLSRCPSCFYNLMNLFCELTCSPRQSQF
Armadillo           ELCPGFFFDNVSLCCDVQQLHTLKDNLQLPLQFLSRCPSCFYNLMNLFCELTCSPRQGQF
Kangaroo rat        ELCPGFFYDNVSVCCDVQQLQTLKSNLQLPLQFLSRCPSCFYNLMSLFCELTCSPQQSQF
Shrew               ELCPGFFFNDVSLCC-VQQLQTLKENLQRPLQFLSRCPSCFYNLMNLFCELTCSPRQSQF
Dolphin             ELCPGFFFGNVSLCCDVQQLRTLKDNLQLPLQFLSRCPSCFYNLMNLFCELTCSPRQSQF
Cow                 ELCPGFFFGNVSLCCDVQQLHTLKDNLQLPLQFLSRCPSCFYNLVNLFCELTCSPRQSQF
Pig                 ELCPGFFFGNVSLCCDVQQLRTLKDNLQLPLQFLSRCPSCFYNLMNLFCELTCSPRQSQF
Weasel              XXXXXXXXXXXXXXXXXXXXXXXXXXXXXXXXXXXXXXXXXXXXXXXXXXXXXXXXXXXX
Panda               ELCPGFFFDNVSVCCDVQQLRTLKDSLQLPLQFLSRCPSCFYNLMNLFCELTCSPRQSQF
Dog                 ELCPGLFFDNVSVCCDVQQLRTLKDSLQLPLQFLSRCPSCFYNLMNLFCELTCSPQQSQF
Cat                 ELCPGFFFDNVSLCCDVQQLRTLKDNLALPLQXXXXCPSCFYNLVNLFCELTCSPRQSQF
Microbat            ELCPGLFFGNVSLCCDVQQLRTLKDNLQLPLQFLSRCPSCFYNLMNLFCELTCSPRQSQF
Horse               ELCPGLFFGNVSLCCDVQQLQTLKDNLQLPLQFLSRCPSCFYNLMNLFCELTCSPQQSQF
Mouse lemur         EHCPGFFFDNVSLCCDVQQLRTLKDNLQLPLQFLSRCPSCFYNLLTLFCELTCSPRQSQF
```

Pika	ELCPGFFFDNVSLCCDVQQLRTLKGNLQLPLQFLSRCPSCFYNLMNLFCELTCSPRQSQF
Rabbit	ELCPGFFFDNVSLCCDVQQLQTLKDNLQLPLQFLSRCPSCFYNLMNLFCELTCSPRQSQF
Orangutan	ELCPGFFFGNVSLCCDVQQLQTLKDNLQLPLQFLSRCPSCFYNLLNLFCELTCSPRQSQF
Human	ELCPGFFFGNVSLCCDVRQLQTLKDNLQLPLQFLSRCPSCFYNLLNLFCELTCSPRQSQF
Chimpanzee	ELCPGFFFGNVSLCCDVQQLQTLKDNLQLPLQFLSRCPSCFYNLLNLFCELTCSPRQSQF
Gorilla	ELCPGFFFGNVSLCCDVRQLQTLKDNLQLPLQFLSRCPSCFYNLLNLFCELTCSPRQSQF
Marmoset	ELCPGFFFDNVSLCCDVQQLQTLKDSLQLPLQFLSRCPSCFYNLLNLFCELTCSPRQSQF
Macaque	ELCPGFFFGNVSLCCDVQQLQTLKDNLQLPLQFLSRCPSCFYNLLNLFCELTCSPRQSQF
Gibbon	XXXXXXXXXXXXXXXXXXXXXXXXXXXXXXXXXXXXXXXXXXXXXXXXXXXXXXXXXXXX
Baboon	ELCPGFFFGNVSLCCDIQQLQTLKDNLQLPLQFLSRCPSCFYNLLNLFCELTCSPRQSQF
Bushbaby	ELCPGFFFDNVSLCCDVQQLQTLKDNLQLPLQFLSRCPSCFYNLLNLFCELTCSPRQSQF
Tarsier	ELCPGFLYDNVSLCCDVQQLQTLKDNLQLPLQFLSRCPSCFYNLMNLFCELTCSPQQGRF
Mouse	ELCPGLFFDNVSLCCDIQQLQTLKSNLQLPLQFLSRCPSCFYNLMTLFCELTCSPHQSQF
Rat	ELCPGLFFGNVSLCCDVQQLQTLKSNLQLPLQFLSRCPSCFYNLMTLFCELTCSPHQSQF
Hamster	ELCPGFFFGNVSLCCDVQQLQTLKSNLQLPMQFLSRCPSCFYNLMTLFCELTCSPHQSQF
Guinea pig	ELCPGFFFDNVSLCCDVQQLQTLKDNLQLPLQFLSRCPSCFYNLMNLFCELTCSPHQSQF
Opossum	ELCPGYFFDDVRLCCDVQQLQTLKSNLQLPLQFLSRCPSCFYNLMNLFCELTCSPRQSQF
Tasmanian devil	XXXXXXXXXXXXXXXXXXXXXXXXXXXLPLGFSFRCPSCFYNLMNLFCELTCSPRQSQF
Platypus	ELCPGLFFGNVSLCCDVQQLHTLKDKLQLPLQFLSRCPSCFYNLMNLFCELTCSPYQGQF
Elephant	ELCPGFFFDNVSLCCDVQQLRTLKDNLQLPLQFLSRCPSCFYNLMNLFCELTCSPQQSQF
Wallaby	ELCPGFFFGNVSLCCDVQQLQTLKNNLQLPLQFLSRCPSCFYNLMNLFCELTCSPRQSWF
Sloth	XXXXXXXXXXXXXXXXXXXXXXXXXXXXXXXXXXXXXXXXXXXXXXXXXXXXXXXXXXXX
Hedgehog	ELCPGFFFDNVSLCCDVQQLQTLKNSLQLPLQFLSRCPSCFYNLMNLFCELTCSHRQSQF
Squirrel	ELCPGFFFDNVSLCCDVQQLQTLKDNLQLPLQFLSXXXXXXXXXXXXXXXXXXXXXXXXX

Tenrec	LNVTATGDYVDPATNQTKTNVEELQYYIGERFANAMYNACRDVEAPSSNEKALGLLCGKD
Macrobat	LNVTATEDYFDPVINQTKTNVKELQYYVGESFANAMYNACRDVEAPSSNDKALGLLCGKD
Hyrax	LNVTATEDYVDPSTNQTKTNVEELQYCIGESFASXXXXXXXXXXXXXXXXXXXXXXXXXX
Tree shrew	LNVTATEDYVDPVTNQTKTNVEELQYYVGQSFANXXXXXXXXXXXXXXXXXXXXXXXXXX
Armadillo	LNVTATEDYVDPVTNQTKINVKELEYYIGESFANAMYNACRDVEAPSSNDKALGLLCGKD
Kangaroo rat	LNVTETEDIVDPVTNQTKTNVKALEYYIGQSFANAMYNACRDVEAPSSNDKALGLLCGKD
Shrew	LNVTATGEYVDPVTNQTKRNVIELQYFIGESFANAMYNACRDVEAPSSNDKALGLLCGKE
Dolphin	LNVTAVEDYVDPVTNQTKTNVKELQYYVGESFANAMYNACRDVEAPSSNEKALGLLCGKE
Cow	LNVTATEDYVDPATNQTKTNVKELQYYVGESFANAMYNSCRDVEAPSSNEKALGLLCGRE
Pig	LNVTATEDYVDPVTNQTKTNVKELEYYVGETFANAMYNACRDVEAPSSNEKALGLLCGRE
Weasel	XXXXXXXXXXXXXXXXXXXXXXXXXXXXXXXXXXXXXXXXXXXXXXXXXXXXXXXXXXXX
Panda	LNVTESEDYIDPVTNQTKTNVKELQYYVGESFANAMYNACRDVEAPSSNDKALGLLCGKD
Dog	LNVTETEDYVDPVTNQTKTNVKELQYYVGESFANAMYNACRDVEAPSSNDKALGLLCGKE
Cat	LNVTATEDYVDPVTNQTKTNVKELQYYIGESFANAMYNACRDVEAPSSNDKALGLLCGKD
Microbat	LNVTETEEYVDPVTHENKTNVKELQYYVGESFANAMYNACRDVEAPSSNDKALGLLCGKE
Horse	LNVTATEDYVDPVTNQTKTNVIELQYYVGESFANAMYNACRDVEAPSSNDKALGILCGKE
Mouse lemur	LNVTATEDYVDPVTNQTRTNVKELQYYVGQSFANAMYNACRDVEAPSSNDKALGLLCGKD
Pika	LNVTATEEYVDPATNQTKTNIKELQYYIGQRFADAMYNACRDVEAPSSNDKALGLLCGKD
Rabbit	LNVTATEAYVDPATNQTKTNVKELQYYIGQRFADAMYNACRDVEAPSSNDKALGLLCGKD
Orangutan	LNVTATEDYVDPVTNQTKTNVKELQYYVGQSFANAMYNACRDVEAPSSNDKALGLLCGKD
Human	LNVTATEDYVDPVTNQTKTNVKELQYYVGQSFANAMYNACRDVEAPSSNDKALGLLCGKD
Chimpanzee	LNVTATEDYVDPVTNQTKTNVKELQYYVGQSFANAMYNACRDVEAPSSNDKALGLLCGKD
Gorilla	LNVTATEDYVDPVTNQTKTNVKELQYYVGQSFANAMYNACRDVEAPSSNDKALGLLCGKD
Marmoset	LNVTATEDYVDPVTNQTKTNVKELQYYVGQSFANAMYNACRDVEAPSXXXXXXXXXXXXX
Macaque	LNVTATEDYVDPVTNQTKTNVKELQYFVGQSFANAMYNACRDVEAPSSNDKALGLLCGKD
Gibbon	XXXXXXXXXXXXXXXXXXXXXXXXXXXXXXXXXXXXXXXXXXXXXXXXXXXXXXXXXXXX
Baboon	LNVTATEDYVDPVTNQTKTNVKELQYFVGQSFANVMYNACRDVEAPSSNDKALELLCGKD
Bushbaby	LNVTATEDYVDPVTNQTRTNVKELQYYIGQTFANAMYNACRDVQAPSSNEKALGLLCGKD
Tarsier	LNVTATKDYVDPVTNQTKTNVVELQYYVGQSFANAMYDACRDVEAPSSNDKALGLLCGKD
Mouse	LNVTATEDYFDPKTQENKTNVKELEYFVGQSFANAMYNACRDVEAPSSNEKALGLLCGRD
Rat	LNVTATEDYFDPETRENKTNVKELEYYVGRSFANAMYNACRDVEAPSSNEKALGLLCGRD
Hamster	LNVTATEDYVDPKTQENKTNVKELEYYIGQSFANEMYNACRDVEAPASNEKALGILCGKD
Guinea pig	LNVTATEDYIDPDTNQTRTNVKELQYYIGQSFANAMYNACRDVEAPSSNDKALGLLCGRD
Opossum	LNVTSTQSFIDPTTNETKTNIDGLQYYIGQSFADAMYNACHDVEAPSSNDKALGLMCGKD
Tasmanian devil	LNVTATKSYIDPTTNETKTNVEELQYYIGQSFADAMYNACRDVESPSSNDKALGLLCGKD
Platypus	LNVTTTGDFVDPVSNITKKNVETL-YYIGKSFADAMYGACRDVEAPSSNDKALGLLCGRD
Elephant	LNVTATNDYVDPVTNETKTNVEELQYYIGDSFANAMYNACRDVEAPSSNDKALGLLCGKD
Wallaby	LNVTDTESHVDPTTNETRINVKGLQYYIGQSFADAMYNACQDVEAPSSNDKALGLLCGKD
Sloth	XXXXXXXXXXXXXXXXXXXXXXXXXXXXXXXXXXXXXXXXXXXXXXXXXXXXXXXXXXXX
Hedgehog	LNITATEDYVDPVTNQTKTNVKELQYYIGETFANAMYNACQDVEAPSSNDRALGLMCGRD

```
Squirrel            XXXXXXXXXXXXXXXXXXXXXXXXXXXXXXXXXXXMYNACRDVEAPSSNDKALGLLCGRD

                    182                               215
Tenrec              AEACNATNWIEYMFNKDNGQAPFTITPVFSDVPLLGMEPMNNATKGCDEAVDEVMGPCSC
Macrobat            AKDCNATNWIEYMFNKDNGQAPFTITPIFSDLPAHGMKPMNNATKGCDESVDEVTGPCSC
Hyrax               XXXXXXXXXXXXXXXXXXXXXXXXXXXXXXXLPVLGMEPMNNATKDCNEAVDEVTGPCSC
Tree shrew          XXXXXXXXXXXXXXXXXXXXXXXXXXXXXXXLPVHGMEPMNNATKACSESVDEVTGPCSC
Armadillo           ASACNATNWIEYMFNKDNGQAPFTITPIFSDLPAHGMEPMNNATKGCNESVDEVTGPCSC
Kangaroo rat        ASACNATNWIEYMFSKDNGQTPFTITPIFSXXXXXXXXXXXXXXXXXXXXXXXXXXXXXX
Shrew               AEACNATNWIEYMFNKDNGQAPFTITPIFSXXXXXXXXXXXXXXXXXXXXXXXXXXXXXX
Dolphin             AEACNATNWIEYMFNKDNGQAPFTITPIFSDLPAHEMEPMNNATKGCNESVDEVTGPCSC
Cow                 ASACNATNWIEYMFNKDNGQAPFTITPVFSDLPTHGMEPMNNATKGCDESVDEVTGPCSC
Pig                 AQACNATNWIEYMFNKDNGQAPFTITPIFSDLPTHGMEPMNNATKGCDESVDEVTGPCSC
Weasel              XXXXXXXXXXXXXXXXXXXXXXXXXXXXXXXXXXXXXXXXXXXXXXXXXXXXXXXXXXXX
Panda               AEACNATNWIEYMFNKDNGQAPFTITPIFSDLPAHAMEPMNNATKGCDESVDEVTGPCSC
Dog                 AEACNATNWIEYMFNKDNGQAPFTIIPIFSDLPAHGMKPMNNATKGCDEPVDEVTAPCSC
Cat                 AEACNATNWIEYMFSKDNGQAPFTITPIFSDLPTHGMEPMNNATKGCDESVDEVTGPCSC
Microbat            AAACNATNWIEYMFNKDNGQAPFTITPVFSDLPAHGMEPMNNATKGCAEPVDEVTGPCSC
Horse               AEACNATNWIEYMFNKNNGQAPFTITPIFSDFPAHGMEPMNNATKGCNESVDEVTGPCSC
Mouse lemur         AAACNATNWIEYMFNKDNGQAPFTITPIFSDLPLQGMEPMNNATKGCNESVDEGTGPCSC
Pika                ASACNATNWIEYMFNKDNGQAPFTITPIFSDLPVSGMEPMNNATKGCNEAVDEVTAPCSC
Rabbit              ANACNATNWIEYMFDKNNGQAPFTITPIFSDLPVRGMEPMNNATKGCNEAVDEVTGPCSC
Orangutan           ADACNATNWIEYMFNKDNGQAPFTITPVFSDFPVHGMEPMNNATKGCDESVDEVTAPCSC
Human               ADACNATNWIEYMFNKDNGQAPFTITPVFSDFPVHGMEPMNNATKGCDESVDEVTAPCSC
Chimpanzee          ADACNATNWIEYMFNKDNGQAPFTITPVFSDFPVHGMEPMNNATKGCDESVDEVTAPCSC
Gorilla             ADACNATNWIEYMFNKDNGQAPFTITPVFSDFPVHGMEPMSNATKGCDESVDEVTAPCSC
Marmoset            XXXXXXXXXXXXXXXXXXXXXXXXXXXXXXDFPVHGMEPMNNATKGCDESVDEATAPCSC
Macaque             XXXXXXXXXXXXXXXXXXXXXXXXXXXXXXDFPVHGMEPMNNATKGCDESVDEVTAPCSC
Gibbon              XXXXXXXXXXXXXXXXXXXXXXXXXXXXXXDFPVHGMEPMNNATKGCDESVDEVTAPCSC
Baboon              ADACNATNWIEYMFNMDNGQAPFTITPVFSDFPVHGMEPMNNATKGCDESVDEVTAPCSC
Bushbaby            AAACNATNWIEYMFNKDNGQAPFTITPVFSDLPAHGMEPMNNATKGCNESVDEVTGPCSC
Tarsier             ADACNATNWIEYMFNKDNGQAPFTITPIFSDLPVHGMEPMNNATKGCNESVDEVTGPCSC
Mouse               ARACNATNWIEYMFNKDNGQAPFTIIPVFSDLSILGMEPMRNATKGCNESVDEVTGPCSC
Rat                 ARACNATNWIEYMFNKDNGQAPFTIIPVFSDLSVLGMEPMRNATKGCNESVDEVTGPCSC
Hamster             ARACNATNWIEYMFNKNNGQAPFTITPIFSDLPILGMEPMRNATKGCNESVDEVTGPCSC
Guinea pig          ADTCNATNWIEYMFNKDNGQAPFTITPIFSDLPIHGMEPMRNATKACNESVDEVTGPCSC
Opossum             AKDCNATNWIEYMFNKDNGQAPFTITPIFSDIPLYGMQPMNNATKGCNESVDDVTGPCSC
Tasmanian devil     AKDCNATNWIEYMFNKNNGQAPFNIIPIFSDNSLYKMEPMNNATKGCNESVDDVTGPCSC
Platypus            AKDCNATNWIEYMFNKDNGQAPFTITPIFSDVSIRGMIPMSNATRGCNESVDETTGPCSC
Elephant            AKACNATNWIEYMFSKDNGQTPFTITPVFSDLPVLGMEPMNNATKGCDEAVDEVTGPCSC
Wallaby             AKDCNATNWIQYMFNKDNGQAPFTITPIFSDIPVYGMEPMNNATKSCNESVDDVTEPCSC
Sloth               XXXXXXXXXXXXXXXXXXXXXXXXXXXXXXXXXXXXXXXXXXXXXXXXXXXXXXXXXXXX
Hedgehog            ASDCNATNWIEYMFSKDNGQAPFTIIPIFSDFPTHGMEPMNNATKGCDESVDEVTGPCSC
Squirrel            ADACNATNWIEYMFDKNNGQAPFTITPIFSDLPVQGMEPMNNATKGCNESVDEVTGPCSC


Tenrec              QDCSIVCGPKPQPPPPPAPWIILGLDAMCVIMWFSYMVFLLIFFGAFFAVWCYRKRYFVS
Macrobat            QDCSAVCGPEPKPPPPPVPWRILGLDAMYVIMWTTYMAFLLVFFGAFFAVWCYRKRYFVS
Hyrax               QDCSAVCGPKPQPPPPPTPWMIFGLDAMYVVMWLTYMAFLLVFFGAFFAVWCYRKRYFVS
Tree shrew          QDCSVMCGPKPQPPPPPVPWRIFGLDAMYVVMWITYMAFLLVFFGAFFAVWCYRKRYFVS
Armadillo           QDCSLTCGPKPQPPPPPAPWVILGLDAMFVIMWITYMAFLLVFFGAFFAVWCYRKRYFVS
Kangaroo rat        XXXXXXXXXXXXXXXXXXXXXXXXXXXXXXXXXXXXXXXXXXXXXXXXXXXXXXKRYFVF
Shrew               XXXXXXXXXXXXXXXXXXXXXXXXXXXXXXXXXXXXXXXXXXXXXXXXXXXXXXKRYFVS
Dolphin             QDCSIVCGPKPQPPAPPVPWRILGLDAMYVIMWSTYMAFLLVFFGAFFAVWCYRKRYFVS
Cow                 QDCSAVCGPKPQPPPPPVPWRILGLDAMYVIMWSTYMAFLLVFFGAFFAVWCYRKRYFVS
Pig                 QDCSIVCGPKPQPPPPPVPWRILGLDAMYVIMWSSYMAFLIVFFGAFFAVWCYRKRYFVS
Weasel              XXXXXXXXXXXXXXXXXXXXXXXXXXXXXXXXXXXXXXXXXXXXXXXXXXXXXXXXXXXX
Panda               QDCSAVCGPKPQPPPPPAPWRILGLDAMYVIMWTTYMAFLLVFFGAFFAVWCYRKRYFVS
Dog                 QDCSVVCGPKPQPPPAPAPWRILGLDAMYVIMWITYMAFLLMFFGAFFAVWCYRKRYFVS
Cat                 QDCSIVCGPKPQPPPPPVPWRILGLDAMYVIMWITYMAFLLVFFGAFFALWCYRKRYFVS
Microbat            QDCSAVCGPRPQPPPPPVPWRIWGLDAMYVVMWATYMAFLLMFFGAIFAVWCYRKRYLVS
Horse               QDCSVVCGPKPQPPPAPAPWRILGLDAMYVIMWTTYMAFLLMFFGAFFAVWCYRKRYFVS
Mouse lemur         QDCSATCGPKPQPPPPLVPWRLFGLDAMYVIMWLTYMAFLLVFFGAFFAVWCYXXXXXXX
Pika                QDCSAMCGPKPQPPPPPVPWRIWGLDAMYVIMWITYMAFLFVXXXXXXXXXXXXXXXXXX
Rabbit              QDCSVVCGPKPQPPPPPIPWRIFGLDAMYVIMWITYMAFLFMFFGTFFAVWCYRKRYFVS
Orangutan           QDCSIVCGPKPQPPPPPAPWTILGLDAMYVIMWITYMVFLLVFFGAFFAVWCYRKRYFVS
```

```
Human             QDCSIVCGPKPQPPPPPAPWTILGLDAMYVIMWITYMAFLLVFFGAFFAVWCYRKRYFVS
Chimpanzee        QDCSIVCGPKPQPPPPPAPWTILGLDAMYVIMWITYMAFLLVFFGAFFAVWCYRKRYFVS
Gorilla           QDCSIVCGPKPQPPPPPAHWTILGLDAMYVIMWITYMAFLLVFFGAFFAVWCYRKRYFVS
Marmoset          QDCSIVCGPKPQPPPPPAPWMILGLDAMYVIMWITYMAFLLMFFGAFFAVWCYRKRYFVS
Macaque           QDCSIVCGPKPQPPPPPAPWTILGLDAMYVIMWITYMAFLLVFFGAFFAVWCYRKRYFVS
Gibbon            QDCSIVCGPKPQPPPPPAPWMILGLDAMYVIMWITYMAFLLVFFGAFFAVWCYRKRYFVS
Baboon            QDCSIVCGPSPSPRRPPAPWTILGLDAMYVIMWITYMGFLLVFFGAFFAVWCYRKRYFVS
Bushbaby          QDCSATCGPKPQPPPPPVPWRLFGLDAMYVIMWITYMAFLLVFFGAFFAVWCYRKRYFVS
Tarsier           QDCSVVCGPKPQPPPPPVPWRIFGLDAMYVIMWITYMAFLLVFFGAFFAVWCYRKRYFVS
Mouse             QDCSIVCGPKPQPPPPPMPWRIWGLDAMYVIMWVTYVAFLFVFFGALLAVWCHRRRYFVS
Rat               QDCSAVCGPKPQPPPPPVPWRILGLDAMYVIMWVAYMAFLVLFFGGLLAVWCHRRRYFVS
Hamster           QDCSIVCGPKPQPPPTPVPWRILGLDAMYVIMWVTYMAFLFIFFGGLLAVWCHRRRYFVS
Guinea pig        QDCSIVC----------VPWRLLGWDAMYVIMWITYMAFLVVFFGACFAVWCYRKRYFVS
Opossum           QDCSITCGPKPQPPPPPTPWIILGLDAMYVIMWIFYMGFLLLFFGMFFIIWCYRKRYFVS
Tasmanian devil   QDCSITCGPKPQPPPPPVPWLIFGLDAMYVIMWIFYMGFLLVFFGMFFIVWCYXXXXXXX
Platypus          QDCSIVCGPKPQPLPPPVPWRIFGLDAMYVIMWLSYMGFLFVFFGAFFGVWCYRKRYFVS
Elephant          QDCSVVCGPKPQPPPPPAPWIIFGLDAMYVIMWITYMAFVLVFFGSFFAVWCYRKRYFVS
Wallaby           QDCSITCGPKPQPPPTPAPWLILGLDAMYVIMWIFYMAFLLVFFGMFFIVWCYRKRYFVS
Sloth             XXXXXXXXXXXXXXXXXXXXXXXXXXXXXXXXXXXXXXXXXXXXXXXXXXXXXKQYFVS
Hedgehog          QDCSVVCGPKPQPPPPPIPWRILGLDAMYVIMWATYMAFLLVFFGVFFAMWCYRKRYFVS
Squirrel          QDCSIVCGPKPQPPAPPIPWRIFGLDAMYVIMWITYMAFLFVFFGAFFALWCYXXXXXXX


Tenrec            EYTPIDSNIAYSVNASDKGEASCCDQLGAAFEGGLRRLFTQWGAFCVRNPGCVIFFSLVF
Macrobat          EYTPIDSNIAFSVNARDKGEASCCDPLGAAFEGCLRRLFTQWGSFCVRNPGCVIFFSLVF
Hyrax             EYTPIDSNIAFSVNTSDKGAASCCGPLGAAFESSLRRLFTQWGAFCVRNPGCVVFFSLVF
Tree shrew        EYTPIDSNIAYSVNASDKGEASCCDPLGAAFEGCLKRLFTQWGSFCVRNPGSIIFFSLVF
Armadillo         EYAPIDSKIASSVNSSNKGEASCCDALSVTLEGFMRQLFTRWGSFCFRNPGCVIFFSLVF
Kangaroo rat      EYTPIDSNIAFSVNSSDKGDTSCCDPLGAAFEGCLKRLFTQWGAFCVRNPXXXXXXSLIL
Shrew             EYTPIDSNIAFSVNASDKGEASCCDPLGAAFEGCLKQLFTQWGSFCVRNPGVILFFSLAF
Dolphin           EYTPIDGNIAFSVNASDKGGPSCCDSLGAAFEARLQRLFTRWGLFCVQHPGCIVFFSV-F
Cow               EFTPIDGNIPFSINASDKGGPTCCDPLGAAFEAHLRRLFEWWGSFCVRHPGCVVFFSVAF
Pig               EYTPIDGNIAFSVNSSDKGQAFCCDPLGAAFERGLRRLFAQWGAFCVRHPGCVVFFSLAF
Weasel            XXXXXXXXXXXXXXXXXXXXXXXXXXXXXXXXXXXXXXXXXXXXXXXXXXXXXXXXXXXX
Panda             EYTPIDSNIAFSVNASDKGEASCCDALAAAFEGYLRRLFSRWGSFCVRNPGCIIFFSLAF
Dog               EYTPIDSNIAFSVNAGDTGEASCCDALGAAFEGCLRRLFTQWGSFCIRNPGCIIFFSLAF
Cat               EYTPIDSNIAFSVNANDRGEASCCDALGAAFEGCLRRLFSQWGSFCVRNPGPIIFFSLAF
Microbat          EYTPIDSSIAFPMSVSDTGEASCCDPLGAAFEGCLRRLFTRWGSFCVRNPGCVIFSSLAF
Horse             EYTPIDSNIAFSVNASDKGEAACCDPLGAAFEGCLRRLFARWGSFCVRNPGCVVVFSLAF
Mouse lemur       XXXXXXXXXXXXXXXXXXXXXXXXXXXXXXXXXXXXXXXXXXXXXXXXXXXXXXXXXXXX
Pika              XXXXXXXXXXXXXXXXXXXXXXXXXXXXXXXXXXXXXXXXXXXXXXXXXXXXXXXXXXXX
Rabbit            EYTPIDSNIAFSVNTSDKGEASCCDLLGAAFEGCLRRLFTRWGSFCVRNPGCVIFFSLGF
Orangutan         EYTPIDSNIAFSVNASDKGEASCCDPVSAAFEGCLRRLFTRWGSFCVRNPGCVIFFSLVF
Human             EYTPIDSNIAFSVNASDKGEASCCDPVSAAFEGCLRRLFTRWGSFCVRNPGCVIFFSLVF
Chimpanzee        EYTPIDSNIAFSVNASDKGEASCCDPVSAAFEGCLRRLFTRWGSFCVRNPGCVIFFSLVF
Gorilla           EYTPIDSNIAFSVNASDKGEASCCDPVSAAFEGCLRRLFTRWGSFCVRNPGCVIFFSLVF
Marmoset          EYTPIDSNIAFSVNASDKGEVSCCDPVSAAFEGCLRQLFTRWGSFCVRNPGCVIFFSLVF
Macaque           EYTPIDSNIAFSVNASDKGEASCCDPVSAAFEGCLRRLFTRWGSFCVRNPGCVIFFSLVF
Gibbon            EYTPIDSNIAFSVNASDKGEASCCDPVSAAFEGCLRRLFTRWGSFCVRNPGCVIFFSLVF
Baboon            EYTPIDSNIAFSVNASDKGEASCCDPVSAAFEGCLRRLFTRWGSFCVRNPGCVIFFSLVF
Bushbaby          EYTPIDSSIAFSVNGSDKGEASCCDPVGAAFEGCLRHLFTRWGSFCVRNPGCVIFFSLVF
Tarsier           EYTPIDSNIAFSVNSSDKGEVSCCDPLGVAFEGCLRRLYTWWG---FLNPGCVIFFSLVF
Mouse             EYTPIDSNIAFSVNSSDKGEASCCDPLGAAFDDCLRRMFTKWGAFCVRNPTCIIFFSLAF
Rat               EYTPIDSNIAFSSNSSDKGEASCCDPLGVAFDDCLRRMFTKWGAFCVRNPTCIIFFSLVF
Hamster           EYTPIDSNIAFSSNTSDKGEASCCDPLGAAFDDCLRRMFTKWGAFCVRNPTCVIFFSLVF
Guinea pig        EYTPIDSNMALGGDTSDKGEIACCDPLGTCFEGCLRHLFTRWGAFCVQRPGYVIFFSLVF
Opossum           EYTPIDGNIAFSINASDRGEASCCDQLGVAFEGFLTQVFTRWGSFCVRKPVLVIFLSLVF
Tasmanian devil   XXXXXXXXXXXXXXXXXXGEASCGDRLGAVFESCLTQGFTRWGSFCVRNPVPVIICSLFF
Platypus          EYAPIDSNIAFSVNASDKGEASRCERLGEMFESGLRWAFSRWGSLCVRHPLPVVVASLAF
Elephant          EYTPIDSNMAFSVNASDKGEASCCDPLGAAFEGCLRRLFTRWGVFCVRNPGCVVFFSLVF
Wallaby           EYTPIDGNIAFSVNASDRGEASCCEQLGAAFEGCLIKGFTQWGSFCVRNP-CVIFCSLVF
Sloth             EYTPIDSNIAFSVNASDQXXXXXXXXXXXXXXXXXXXXXXXXXXXXXXXXXXXXXXXXXX
Hedgehog          EYTPIDSNIAFSVNASDKGEVSCCDPVSAAFENCLRQLFSQWGSFCVRNPGCIIFFSVAF
Squirrel          XXXXXXXXXXXXXXXXXXXEASCCDRLGAAFEGCLRRFFTQWGSFCVRNPGCVIFFSVVF
```

416,417

```
Tenrec              IGVCSLGLMFVQVTTNPVDLWSSPSSQARHEKEYFDTHFGPFFRTEQLIIRAPQTRKHTY
Macrobat            IATCSSGLVFVQVTTNPVHLWSAPSSQARLEKEYFDTHFGPFFRTEQLIIRAPHTATHTY
Hyrax               IGVCCSGLVFLRVTTNPIDLWSAPSSQTRQEKEYFDTHFGPFFRIEQLIIQAPHTAKHTY
Tree shrew          IAACSSGLVFVRVTTNPVDLWSAPNSQARLEKEYFDAHFGPFFRTEQLIIQAPHTDRHTY
Armadillo           IGVCSSGLVFVRVTTNPVDLWSAPSSQARLEKEYFDAHFGPFFRTEQLIIRAPHTARHTY
Kangaroo rat        XXXXXXXXXXXXXXXXXXXWSSPNSQARLEKEYYDTHFGPFFRTEQIIIQAPHTSVHIY
Shrew               IGACASGLVFVRITTNPVELWSAPSSQARQEKQYFDTHFGPFFRTEQLIIRAPHTKAHTY
Dolphin             IAACSSGLVFVQVTTDPVDLWSAPGSQARREKEYFDTHFGPFFRTEQLIIQAPLTQPHMY
Cow                 IAACSSGLVFIQVTTDPVDLWSAPGSQARLEKEYFDTHFGPFFRTEQLIIRAPHTPPHIY
Pig                 IVACSSGLVFIRVTTDPVDLWSAPGSQARREKEYFDTHFGPFFRTEQLIIRATNNQSHIY
Weasel              XXXXXXXXXXXXXXXXXXXXXXXXXXXXXXXXXXXXXXXXXXXXXXXXXXXXXXXXXXXX
Panda               IAACSSGLVFVRVTTNPVDLWSAPGSQARLEKEYFDTHFGPFFRTEQLIIQAPNTSAHTY
Dog                 IAACSSGLVFSRVTTNPVDLWSAPGSQARLEKEYFDAHFGPFFRTEQLIIQAPHTSVHTY
Cat                 IAACSSGLVFVRVTTNPVDLWSAPSSQARLEKEYFDTHFGPFFRTEQLIIQAPHTSAHTY
Microbat            IAACSSGLVFVRITTSPVDLWSAPSSQARLEKEYFDTHFGPFFRTEQLIIQAPHTAVHTY
Horse               IAACSSGLVFVRVTTNPVDLWSAPSSQARLEKEYFDAHFGPFFRTEQLIIRAPHGHVHTY
Mouse lemur         XXXXXXXXXXXVTTIPVDLWSAPASQARLEKEYFDQHFGPFFRTEQLIIRAPLTKKHTY
Pika                XXXXXXXXXXXXXXXXXXXXXXXXXXXXXXXXXFNPHSGLLFRTEQLIIQAPHTDKHIY
Rabbit              IAACSSGLVYVRVTTNPIDLWSAPSSQARQDKEFFDAHFGPFFRTEQLIIRAPHTNKHTY
Orangutan           VTACSSGLVFVRVTTNPVDLWSAPSSQARLEKEYFDQHFGPFFRTEQLIIRAPLTDKHTY
Human               ITACSSGLVFVRVTTNPVDLWSAPSSQARLEKEYFDQHFGPFFRTEQLIIRAPLTDKHIY
Chimpanzee          ITACSSGLVFVWVTTNPVDLWSAPSSQARLEKEYFDQHFGPFFRTEQLIIRAPLTDKHTY
Gorilla             ITACSSGLVFVRVTTNPVDLWSAPSSQARLEKEYFDQHFGPFFRTEQLIIRAPLTDKHTY
Marmoset            IAVCSSGLVFVRVTTNPVDLWSAPSSQARLEKEYFDQHFGPFFRTEQLIIQAPLTDKHTY
Macaque             IAVCSSGLVFVRVTTNPIDLWSAPSSQARLEKEYFDQHFGPFFRTEQLIIRAPLTDKHTY
Gibbon              ITACSSGLVFVRVTTNPVDLWSAPSSQARLEKEYFDQHFGPFFRTEQLIIRAPLTDKHTY
Baboon              IAVCSSGLVFVRVTTNPIDLWSAPSSQARLEKEYFDQHFGPFFRTEQLIIRAPLTDKHTY
Bushbaby            ITTCSSGLVFIRVTTNPVDLWSAPRSQARLEKEYFDQHFGPFFRTEQLIIRAPLTNTHTY
Tarsier             IVVCSSGMVIVRVTTNPVDLWSAPSSQARLEKEYFDQHFGPFFRTEQLIIRAPLTNKHTY
Mouse               ITVCSSGLVFVQVTTNPVELWSAPHSQARLEKEYFDKHFGPFFRTEQLIIQAPNTSVHIY
Rat                 IAACSSGLVFVQVTTNPVELWSAPHSQARLEKEYFDKHFGPFFRTEQLIIQAPNSSVHIY
Hamster             ITACSSGLVFVRVTTNPVELWSAPHSRARLEKEYFDKHFGPFFRMEQLIIQAPNTSEHIY
Guinea pig          IASCSSGLVFVRLTTNPVDLWSAPSSQARLEKEYFDTHFGPFFRTEQLIIQAPHTGKHPY
Opossum             ISVCCSGLVFMRLTTNPVDLWSAPSSQAHLEKEYFDTHFGPFFRTEQLIIRAPHTNPHTY
Tasmanian devil     IAVCSSGLVFMRLATNPVDLWSAPNSQAHLEKEYFDTHFGPFFRTEQLIIQAPHTKGHIY
Platypus            IAACCSGLASVRITTNPVDLWSASGSQARREKEYFDTRFGPFYRTEQLIIRAPNTSRHTF
Elephant            IGVCSSGLVFVRVTTNPIDLWSAPNSQGRREKEYFDMHFGPFFRTEQLIIRSPHTSKHIY
Wallaby             IAACCSGLVFMRLTTNPVDLWSAPNSQAHLEEYFDTHFXXXXXXXXXXXXXXXXXXXXXX
Sloth               XXXXXXXXXXXXXXXXXXXXXXXXXXXXXXXXXXXXXXXXXXXXXXXXXXXXXXXXXXXX
Hedgehog            IAACSSGLVFVQVTTNSVDLWSAP-SQARLEKE-YDTHG-PFFRTXXXXXXXXXXXXHTY
Squirrel            IAACSSGLAFVRVTTNPVDLWSSASSQARQDKEYFDTHFGPFFRTEQLIIRAPNTSKHIY

                   421          434
Tenrec              SPYPSGADVSFGPPLDKEILHQXXXXXXXXXXXXXXXXXXXXXXXXXXXXXXXXXX-XXX
Macrobat            QPYPTETDVPFGPPLDIEILHQXXXXXXXXXXXXXXXXXXXXXXXXXXXXXXXXXX-XXX
Hyrax               QPYPSGADVPFGPPLNIEILHQVLDLQTAIENITASYNNETVTLQDICLAPLSPYN-KNC
Tree shrew          KPYPSGADVPFGPPLDLEILHQVLDLQTAIENITASYNNETVTLQDICLAPLSPYN-KNC
Armadillo           SPYPSGADVPFGPPLNIGILHQVLDLQTAIENISASYNNQTVTLQDICLAPLSPYN-KNC
Kangaroo rat        EPYPSGANVPFGPPLDLEILHQVLDLQTDIENIVAFYNNETVTLQDICVAPLSPYN-KNC
Shrew               EPYPSGSDVPFGPPMDKEILHQVLELQTAIENITASYNNETVTLQDICLTPLSPYN-RNC
Dolphin             HPYPSGADVPFGPPLAVDILHQVLDLQTAIENITASYNNETVTLQDICLAPLSPYN-KHC
Cow                 EPYPSGADVPFGPPLAVDILHQVLDLQTAIESITASYNNETVTLRDICVAPLSPYN-QNC
Pig                 HPYPAGADVPFGPPLSRDILHQVLDLQTAIENITASYNNETVTLQDICLAPLSPYN-KNC
Weasel              XXXXXXXXXXXXXXXXXXXXXXXXXXXXXXXXXXXXXXXXXXXXXXXXXXXXXXXXXXXX
Panda               QPYPSGSDVPFGPPLDIGILHQVLDLQTAIENITAFYNNETVTLQDICVAPLSPYN-KNC
Dog                 QPYPSGSDVPFGPPLDIGILHQVLDLQTAIENITATYNNETVTLQDICVAPLSPYN-KNC
Cat                 QPYPSGSDVPFGPPLDLAILHQVLDLQTAIENITASYNNETVTLQDICVAPLSPYN-KNC
Microbat            EPYPSGSDVPFGPPLDKGILHQVLDLQTAIEHITASHNNETVTLQDICLAPLSPYN-KNC
Horse               EPYPSGADVPFGPPLALEVLHQVLDLQTAIENITASYNNETVTLQDICLAPLSPYN-KNC
Mouse lemur         EPYPSGADVPFGPPLDVEILHQVLDLQTAIENITASYNNETVTLRDICLAPLSPYN-ENC
Pika                QPYPSGADVPFGPPLDKQILHQVLDLQTAIENITASYNNETVTLQDICLAPLAPYN-KNC
Rabbit              QPYPSGADVPFGPPLDKQILHQVLDLQTAIEDITASYNNETVTLQDICLAPLSPYN-KNC
Orangutan           QPYPSGADVPFGPPLDIQILHQVLDLQIAIENITASYNNETVTLQDICLAPLSPYN-TNC
Human               QPYPSGADVPFGPPLDIQILHQVLDLQIAIENITASYDNETVTLQDICLAPLSPYN-TNC
Chimpanzee          QPYPSGADVPFGPPLDIQILHQVLDLQIAIENITASSNNETVTLQDICLAPLSPYN-TNC
Gorilla             QPYPSGADVPFGPPLDIQILHQVLDLQIAIENITASYNNETVTLQDICLAPLSPYN-THC
```

```
Marmoset          QPYPSGADVPFGPPLDIQILHQVLDLQIAIENITASYNNETVTLQDICLAPLSPYN-TNC
Macaque           QPYPSGADVPFGPPLDIQILHQVLDLQIAIENITASYNNETVTLQDICLAPLSPYN-TNC
Gibbon            QPYPSGADVPFGPPLDIQILHQVLDLQIAIENITASYNNETVTLQDICLAPLSPYN-TNC
Baboon            QPYPSGADVPFGPPLDIQILHQVLDLQIAIENITASYNNETVTLQDICLAPLSPYN-TNC
Bushbaby          EPYPSGADVPFGPPLNVEILHQVLDLQTAIENITASYNNETVTLQDICVAPLSPYN-KNC
Tarsier           QPYPSGADVPFGPPLDLEILHQVLDLQTTIENITASYNNGTVTLQDICLAPLSPYN-KNC
Mouse             EPYPAGADVPFGPPLNKEILHQVLDLQIAIESITASYNNETVTLQDICVAPLSPYN-KNC
Rat               EPYPSGADVPFGPPLNKEILHQVLDLQIAIESITASYNNETVTLQDICVAPLSPYN-KNC
Hamster           EPYPSGSDVSFGPPLNKEILHQVLDLQIAIESITTSYNNKTVTLQDICVAPLSPYN-KNC
Guinea pig        SPYPSGADVPFGPPLDKEILHQVLDLQIAIENITAYYNNETVTLGDICLAPLSPYN-KNC
Opossum           EPYPSGADVPFGPALDKGILHQVLDLQTAIENITAFYNNETVLFQDICLAPLSPYN-NNC
Tasmanian devil   QPYPSGADVPFGPPLDKGILHQVLDLQTAIENITASYNNETVTLEDICLAPLSPYN-NNC
Platypus          SPYPSGTDVPFGPPLDKTILHKVLDLQTAIENITASYGNETVRLRDICLAPLAPYN-DNC
Elephant          QPYPSGTDVPFGPPLNIEILHQVLDLQTAIENITALCNNQTVMLRDICLAPLSPYN-KNC
Wallaby           XXXXXXXXXXXXXXXXXXXXXXXXXXXXXXXXXXXXXXXXXXXXXXXXXXXXXXXXXXXX
Sloth             XXXXXXXXXXXXXXXXXXXXXXXXXXXXXXXXXXXXXXXXXXXXXXXXXXXXXXXXXXXX
Hedgehog          QP-PSGSDV-FGPP-DIELHQXXXXXXXXXXXXXXXXXXXXXXXXXXXXXXXXXXXXXXX
Squirrel          QPYPAGADVPFGPPLDKEILHQXXXXXXXXXXXXXXXXXXXXXXXXXXXXXXXXXXXXXX


Tenrec            XXXXXXXXXXXXXXXXXXXXXXXXXXXXXXXXXXXXXXXAPASLNDTSLLHDPCLGTFGG
Macrobat          XXXXXXXXXXXXXXXXXXXXXXXXXXXXXXXXXXXXXXXAPASLNDTSLLHDPCLGTFGG
Hyrax             TILSVLNYFQNSHSVLDHNVGDEFYVYADYHTHFLYCVRAPSSLNDTSLLHDPCLGTFGG
Tree shrew        TIMSVLNYFQNSHSMLDHKIGDDFFVYADYHTHFLYCVRAPASLNDTSLLHDPCLGTFGG
Armadillo         TIMSVLNYFQNSHSVLDHVKGDDFFVYADYHTHFLYCVRAPASLNDTSLLHDPCLGTFGG
Kangaroo rat      TIMSVLNYFQNSHLMLDHKIQDDFYIYADFHTHFLYCVRAPASLNDTSMLHDPCLGTFGG
Shrew             TILSVLNYFQNSHAMLDRKIEDDFFVYADYHTHFLYCVRAPASLNDTSVLHDPCLGTFGG
Dolphin           TIMSVLNYFQNSHSVLDHKVGDYFFVYADYHTHFLYCVRAPASLNDTSLLHDPCLGTFGG
Cow               TILSVLNYFQNSHSVLDHQVGDDFFVYADYHTHFLYCVRAPASLNDTSLLHDPCLGTFGG
Pig               TILSVLNYFQNSHSVLDHQVGDFFFVYADYHTHFLYCVRAPASLNDTSLLHDPCLGTFGG
Weasel            XXXXXXHHHQNSHSVLDHEVGDDFFVYADYHTHLLYCVRAPASLNDTSLLHDPCLGTFGG
Panda             TILSVLNYFQNSHSLLDHKIGDDFFVYADYHTHLLYCVRAPASLNDTSLLHDPCLGTFGG
Dog               TIMSVLNYFQNSHSMLDHKIGDDFYVYADYHTHLLYCVRAPASLNDTSLLHDPCLGTFGG
Cat               TILSVLNYFQNSHSMLDHEIGDDFFVYADYHTHLLYCVRAPASLNDTSLLHDPCLGTFGG
Microbat          TILSVLNYFQNSHSVLDHEIGDDFYTYADYHTHLLYCVRAPASLNDTSLLHDPCLGTFGG
Horse             TIMSVLNYFQNSHSMLDHKVEDDFFVEADYHTHFLYCVRAPASLNDTTLLHDPCLGTFGG
Mouse lemur       TILSVLNYFQNSHSVLDHKVGDDFFVYADYHTHFLYCVRXXXXX-XXXXXXXXXXLTFGG
Pika              TILSVSNYFQNSHSVLDHKKGDDFFVYPDYHTPFXXXXXAPTSLNDTSLLHDPCLGTFGG
Rabbit            TILSVLNYFQNSHSMLDHEQGDDFFVYADYHTHFLYCVRVPASLNDTSLLHDPCLGTFGG
Orangutan         TIMSVLNYFQNSHSVLDHKKGDDFFVYADYHTHFLYCVRAPASLNDTSLLHDPCLGTFGG
Human             TILSVLNYFQNSHSVLDHKKGDDFFVYADYHTHFLYCVRAPASLNDTSLLHDPCLGTFGG
Chimpanzee        TILSVLNYFQNSHSVLDHKKGDDFFVYADYHTHFLYCVRAPASLNDTSLLHDPCLGTFGG
Gorilla           TILSVLNYFQNSHSVLDHKKGDDFFVYADYHTHFLYCVRAPASLNDTSLLHDPCLGTFGG
Marmoset          TIMSVLNYFQNSHSVLDHKIGDDFFVYADYHTHFLYCVRAPASLNDTSLLHDPCLGTFGG
Macaque           TIMSVLNYFQNSHSVLDHKKGDDFFVYADYHTHFLYCVRAPASLNDTSLLHDPCLGTFGG
Gibbon            TIMSVLNYFQNSHSVLDHKKGDDFFVYADYHTHFLYCVRAPASLNDTSLLHDPCLGTFGG
Baboon            TIMSVLNYFQNSHSVLDHKKGDDFFVYADYHTHFLYCVRAPASLNDTSLLHDPCLGTFRG
Bushbaby          TILSVLNYFQNSHSVLDHKIGDDFFVYADYHTHFLYCVRAPASLNDTSLLHDPCLGTFGG
Tarsier           TIMSVLNYFQNSHSVLDHKIGDEFYVYADYHTHFLYCVRXXXXXXXXXXXXXXXXXXXXX
Mouse             TIMSVLNYFQNSHAVLDSQVGDDFYIYADYHTHFLYCVRAPASLNDTSLLHGPCLGTFGG
Rat               TIMSVLNYFQNSHAVLDNQVGDDFFVYADYHTHFLYCVRAPASLNDTSLLHGPCLGTFGG
Hamster           TIISVLNYFQNSHSVLDHQVGDDFYVYADYHTHFLYCVRAPTSLNDTSLLHDPCLGTFGG
Guinea pig        TIFSVLNYFQNSHATLDHIVGDDFFVYADYHTHFLYCVRAPASLNDTSLLHDPCLGTFGG
Opossum           TILSVLNYFQNSHSVLDHTKGDAFFVYADYHTHFLYCTRAPASLNDTSLLHDPCLGTFGG
Tasmanian devil   TILSVLNYFQNSHSVLDHQKGDEFFVYADYHTHFLYCTRAPASLNDTSLLHDSCLGTFGG
Platypus          TIMSLLNFFQNSHSVLDHERGDGFFVYADYHTHFLYCVRAPTSLNDTSLLHDPCLGTFGG
Elephant          TILSVLNYFQNSHSVLDHKVGDEFYTYADYHTHFLYCVRAPTSLNDTSLLHDPCLGTFGG
Wallaby           XXXXXXXXXXXXXXXXXXXXXXXXXVYADYHTHFLYCTRAPASLNDTSLLHDSCLGTFGG
Sloth             XXXXXXXXXXXXXXXXXXXXXXXXXXXXXXXXXXXXXXXAPASLNDTSVLHDPCLGTFGG
Hedgehog          XXXXXXXXXXXXXXXXXXXXXXXXXXXXXXXXXXXXXXXXXXXXXXXXXXXXXXXXXXXX
Squirrel          XXXXXXXXXXXXXXXXXXXXXXXXXXXXXXXXXXXXXXXXXXXXXXXXXXXXXXXXXXXX


Tenrec            PVFPWLVLGGYDAQNYNNATDLVITFPVNNHYNDTDKLQKALAWERXXXXFVKNYKNPNL
Macrobat          PVFPWLVLGGYDDQNYSNATALVITFLVNNYYNNTEKLQRAQAWERXXXXXXXXXXXXXX
Hyrax             PVFPWLVLGGYDDQNYNNATALVITFPVSNYYNDTEKLQRAQAWEREFINFVKNYKNPNL
```

```
Tree shrew          PVFPWLVLGGYDDQNYGNATALVITFPVNNYYNDTEKLQQAQAWEKEFINFVKNYKNPNL
Armadillo           PVFPWLVLGGYDGQNYNNATALVITFPVNNYYNDTEKLQRAQAWEREFINFVKKYKNPNL
Kangaroo rat        PVFPWLVLGGYDDQNYNNATALVITFPVNNYYNDTEKLQKAQAWEKELINFVKNYSNPNL
Shrew               PVFPWLVLGGYDDQNYNNATALVMTFPVNNYYNDTEKLRRTEAWEREFINFVKSYKHPNL
Dolphin             PVFPWLVLGGYDDENYNNATALVITFPVNNYRNDTEKLQKAQAWEREFINFVKNYKNPNL
Cow                 PVFPWLVLGGYDDQNYNNATALVITFPVNNYYNDTEKLQRAQAWEREFINFVQNYENPNL
Pig                 PVFPWLVLGGYDDQNYNNATALVITFPVNNYYNDTEKLQRAQAWESEFINFVKNYKNPNL
Weasel              PVFPWLVLGGYDDQNYNNATALVITFPVNNYYNDTEKLQRAQAWEKEFINFVKNYENPNL
Panda               PVFPWLVLGGYDDQNYNNATALVITFPVNNYYNDTEKLQRAQAWEKEFINFVKNYENPNL
Dog                 PVFPWLVLGGYDDQNYNNATALVITFPVNNYYNDTEKLQRAQAWEKEFINFVKNYENPNL
Cat                 PVFPWLVLGGYDDQNYNNATALVITFPVNNYYNDTERLQKAHVWEKEFINFVKNYKNPNL
Microbat            PVFPWLVLGGYDDQNYNNATALVMTFPVNNYYNDTEKLQRAQAWEREFINFVKNYKNPNL
Horse               PVFPWLVLGGYDDQNYNNATALVITFPVNNYYNDTEKLQRALAWEREFINFVKNYKNPNL
Mouse lemur         PVFPWLVLGGYDDQNYNNATALVITFPVSNYYNDTEKLQRAQAWEKEFINFVKNYKNPNL
Pika                PVFPWLVLGGYDDQNYNNATALVITFPVNNYYNDTEKLQRAQAWEKEFIDFVKNYKNPNL
Rabbit              PVFPWLVLGGYDDQNYNNATALVITFPVSNYYNDTEKLQRAKAWEKEFINFVKNYENPNL
Orangutan           PVFPWLVLGGYDDQNYNNATALVITFPVNNYYNDTEKLQRAQAWEKEFINFVKNYKNLNL
Human               PVFPWLVLGGYDDQNYNNATALVITFPVNNYYNDTEKLQRAQAWEKEFINFVKNYKNPNL
Chimpanzee          PVFPWLVLGGYDDQNYNNATALVITFPVNNYYNDTEKLQRAQAWEKEFINFVKNYKNPNL
Gorilla             PVFPWLVLGGYDDQNYNNATALVITFPVNNYYNDTEKLQRAQAWEKEFINFVKNYKNPNL
Marmoset            PVFPWLVLGGYDDQNYNNATALVITFPVSNYYNDTEKLQRAQAWEKEFINFVKNYKNSNL
Macaque             PVFPWLVLGGYDDQNYNNATALVITFPVNNYYNDTEKLQRAQAWEKEFINLVRNYKNPNL
Gibbon              PVFPWLVLGGYNDQNYNNATALVITFPVNNYYNDTEKLQRAQAWEKEFINFVKSYKNPNL
Baboon              PVFPWLVLGGYDDQNYNNATALVITFPVNNYYNDTEKLQRAQAWEKEFINLVRNYKNPNL
Bushbaby            PVFPWLVLGGYDDENYNNATALVITFPVNNYYNDTEKLQRAQAWEKEFIDFVKNYKNPNL
Tarsier             XXXXXXXXXXXXXQNYNNATALVITFPVNNYYNDTEKLQRAQAWEKELINFVKNYKNPNL
Mouse               PVFPWLVLGGYDDQNYNNATALVITFPVNNYYNDTERLQRAWAWEKEFISFVKNYKNPNL
Rat                 PVFPWLVLGGYDDQNYNNATALVITFPVNNYYNDTEKLQRAWAWEKEFINFVKNYKNPNL
Hamster             PVFPWLVLGGYDDQNYNNATALVITFPVSNYYNDTEKLQRAQAWEKEFIDFVKSYKNPNL
Guinea pig          PVFPWLVLGGYDDQNYNNATALVITFPVNNYYDDPEKLQRAQAWEKEFIHFVSNYKNPNL
Opossum             PIFPWLVLGGYDDQNYNNATALVITLPVNNYYNNTEKLQKAQAWEKEFINFVKNYKNPNL
Tasmanian devil     PIFPWLVLGGYDDQNYNNATALVITLPVNNYYNNTEKLQKAQAWEKEFISFVKNYKNPNL
Platypus            PVFPWLVLGGYDDQNYNNATALVITFPVNNYHNDTEKLRRAEAWEKEFIRFVRDYKDPNL
Elephant            PVFPWLVLGGYDDQNYNNATALVITFPVNNYYNDTEKLQRAQAWEREFINFVKNYENPNL
Wallaby             PIFPWLVLGGYDEQNYNNATALVITLPVNNYYNNTEKVQRAQAWETEFIHFIKNYRNPNL
Sloth               PVFPWLVLGGYDDQNYNNATALVITFPVNNYYNNTEKLQRAQAWEREFINFKN-YKNPNL
Hedgehog            XXXXXXXXXXXXXXXXXXXXXXXXXXXXXXXXXXXXXXXXXXXXXXXXFISFVKDYKNPNL
Squirrel            XXXXXXXXXXXXQNYNNATALVITFPVNNYYNDTEKLQRAQAWEKEFINFVKNYKNPNL

                                                         642
Tenrec              TISFTAERSIEDELNRQSNSDVFTVAISYTVMFVYISIAVGHIRSCHRLMVDSKVSLGIA
Macrobat            XXXXXXXXXXXXXXXXXXXXXXXXXXXXXXXXXXXXXXXXXXXXXXXXXXXXXXXXXXXX
Hyrax               TISFTAERSIEDELNRESRGDVFTVVISYAIMFLYISIALGHIKSCSRLLXXXXXXXXXA
Tree shrew          TISFTSERSIEDELNRESNSDVFTVVISYAIMFLYISLALGHIKSCHRFLVDSKISLGIA
Armadillo           TISFIAERSVEDELDRESNSDVFTIIISYAVMFLYISIALGHIKSLSRFLXXXXXXXXXX
Kangaroo rat        TISFTTERSIEDELNRESNSDVFTIAISYAIMFLYISLALGHIKSCSRLLXXXXXXXXXX
Shrew               TISFTAERSIEDELNRESKSDVYTVVISYAIMFLYISIALGHIKSCRRLLVDSKISLGIA
Dolphin             TISFKAERSIEDEINRESNSDIFTVLISYGIMFLYISIALGHIESCSRFLVDSKISLGIA
Cow                 TISFKAERSIEDELNRESNSDVFTVLISYGVMFLYISIALGHIKSCRRLLVDSKISLGIA
Pig                 TISFMAERSIEDELNRESNSDLFTILISYAIMFLYISIALGHIKSCSRLLVDSKISLGIA
Weasel              TISFTTERSIEDELNRESNGDVFTVLISYAVMFLYISIALGHIKSWSRFLVDSKISLGIA
Panda               TISFTTERSIEDELNRESNGDVFTVLISYAVMFLYISIALGHIKSCSRFLVDSKISLGIA
Dog                 TISFTTERSIEDELNRESNGDVFTVLISYAVMFLYISIALGHIKSCSRFLVDSKISLGIA
Cat                 TISFTTERSIEDELNRESNGDIFTVIISYAIMFLYISIALGHIKSCSRLLVDSKISLGIA
Microbat            TISFTAERSIEDELNRESNSDISTVVTSYAVMFLYISIALGHIQSCSRLLVDSKVSLGIA
Horse               TISFSAERSIEDELNRESNSDVFTVVISYAVMFLYISIALGHIKSCSRLLVDSKISLGVA
Mouse lemur         TISFSAERSIEDELNRESNSDVFTVLISYAIMFLYISLALGHIKSCRRLLVDSKVSLGIA
Pika                TISFTAERSIEDELNRESNSDVFTVVISYAVMFLYISLALGHIKSCRRLLS------GFA
Rabbit              TISFTAERSIEDELDRESNSDVFTVVISYAVMFLYISLALGHIKSCRRFLVDSKVSLGIA
Orangutan           TISFTAERSIEDELNRESDSDVFTIVISYAIMFLYISLALGHIKSCRRLLVDSKVSLGIA
Human               TISFTAERSIEDELNRESDSDVFTVVISYAIMFLYISLALGHIKSCRRLLVDSKVSLGIA
Chimpanzee          SISFTAERSIEDELRRESDSDVFTVVISYAIMFLYISLALGHIKSCRRLLVDSKVSLGIA
Gorilla             TISFTAERSIEDELNRESDSDVFTVVISYAIMFLYISLALGHIKSCRRLLVDSKVSLGIA
Marmoset            TISFTAERSIEDELNRESNSDIFTVIISYAIMFLYISLALGHIKSCRRLLVDSKVSLGIA
Macaque             TISFSAERSIEDELNRESDSDIFTVVISYAIMFLYISLALGHIKSCRRLLVDSKISLGIA
Gibbon              TISFTAERSIEDELNRESDSDVFTVVISYAIMFLYISLALGHIKSCRRLLVDSKVSLGIA
```

```
Baboon            TISFSAERSIEDELNRESDSDIFTVVISYAIMFLYISLALGHIKSCRRLLVDSKISLGIA
Bushbaby          TISFSAERSIEDEINRESNSDLFTIVISYAVMFVYISLALGHIKSCHRLLVDSKVSLGIA
Tarsier           TISFTTER--IEDLNRESNSDVFTVVISYGIMFLYISLALGHIKSCRKXXVDSKISLGIA
Mouse             TISFTAERSIEDELNRESNSDVFTVIISYVVMFLYISLALGHIQSCSRLLVDSKISLGIA
Rat               TISFTAERSIEDELNRESNSDVFTVLISYAVMFLYISLALGHIQSWNRVLVDSKISLGIA
Hamster           TISFIAERSIEDELNRESNSDVFTIAISYAIMFLYISLALGHIKSCSRLLVDSKISLGIA
Guinea pig        TISFTAERSIEDELNRESNSDVFTIVISYAVMFLYISLALGHIRGCCRFLIDSKISLGIV
Opossum           TISFSAERSIEDEINRESNGDVFTVLISYAVMFLYISIALGHIKSCHRFLVDSKISLGIA
Tasmanian devil   TISFSAERSIEDEINRESNGDVVTVLISYAVMFLYISIALGHIKSFHRFLVDSKISLGIA
Platypus          TISFSAERSIEDELNRESNSDIFTVVISYAVMFLYISLALGHIKSFSRLLVDSKISLGIA
Elephant          TISFTAERSIEDELNRESSSDVLTIVISYAIMFFYISVALGHIKSCSRLLVDSKISLGVA
Wallaby           TISFSAERSIEDEINRESNGDVVTILISYGVMFLYISIALGHIRSCHRFLVELKSSLGIA
Sloth             TISFTAERSIEDELDRESNSDIFTIIISYAIMFLYISVALGHIKSCRRFLVDSKISLGIA
Hedgehog          TVSFSAEQSIEDELNRESNSDVFTIMISYGIMFLYISIALGHIKSCSRILVDSKVSLGIA
Squirrel          TISFTTERSIEDELNRESNGDIFTVVISYAIMFLYISLALGXXXXXXXXXVDSKISLGIT


Tenrec            GILIVLSSVACSLGIFSYIGIPLTLIVIEVIPFLVLAVGVDNIFILVQTYQRDERLQGET
Macrobat          XXFIVLSSVACSLGIS-YIGIPLTLIVIEVIPFLVLAIGVDNIFILVQTYQRDERLQGET
Hyrax             GIVIVLSSVVCSLGIFSYIGIPLT-IVIEVIPFLVLAVGVDNIFILVQTYQRDERHQGET
Tree shrew        GILIVLSSVACSLGIFSYIGVPLTLIVIEVIPFLVLAVGVDNIFILVQTYQRDERLHGET
Armadillo         XXXXXXXXXXXXXXXXXXXXXXXXXXXXXXXXXXXXXXXXXXXXXXXXXXXXXXXXXXXX
Kangaroo rat      XXXXXXXXXXXXXXXXXXXXXXXXXXXXXXXXXXXXXXXXXXXXXXXXXXXXXRDERLQGET
Shrew             GILIVLSSVACSLGIFSFLGIPLTLIVIEVIPFLVLAVGVDNIFILVQTYQRDERLQGES
Dolphin           GILIVLSSVACSLGIFSYIGIPFTLIVIEVIPFLVLAVGVDNIFILVQTYQRDERLQGET
Cow               GVLIVLSSVACSLGIFSYIGVPLTLIVIEVIPFLVLAVGVDNIFILVQTYQRDERLQGET
Pig               GILIVLSSVACSLGIFSYIGVPLTLIVIEVIPFLVLAVGVDNIFILVQTYQRDERLQGET
Weasel            GILIVLSSVACSLGIFSYVGIPLTLIVIEVIPFLVLAVGVDNIFILVQTYQRDERLQGET
Panda             GILIVLSSVACSLGIFSYIGIPLTLIVIEVIPFLVLAVGVDNIFILVQTYQRDERLQGET
Dog               GILIVLSSVMCSLGIFSYFGIPLTLIVIEVIPFLVLAVGVDNIFILVQTYQRDERLQGET
Cat               GILIVLSSVACSLGIFSYVGIPLTLIVIEVIPFLVLAVGVDNIFILVQTYQRDERLHGET
Microbat          GIFIVLSSVACSLGIFSYVGVPLTLIVIEVIPFLVLAVGVDNIFILVQTYQRDERLEGET
Horse             GILIVLSSVACSLGIFSYIGIPLTLIVIEVIPFLVLAVGVDNIFILVQTYQRDERLQGET
Mouse lemur       GILIVLSSVACSLGVFSYIGIPLTLIVIEVIPFLVLAVGVDNIFILVQTYQRDERLQGET
Pika              RLLMGLRSVDCSLGIFSYFGVPLTLLVIEIISFPLAVA--NNIFILVQTYQRDERLQGET
Rabbit            GILIVLSSVACSLGIFSYIGIPLTLIVIEVIPFLVLAVGVDNIFILVQTYQRDERLQGET
Orangutan         GILIVLSSVACSLGVFSYIGLPLTLIVIEVIPFLVLAVGVDNIFILVQAYQRDERLQGET
Human             GILIVLSSVACSLGVFSYIGLPLTLIVIEVIPFLVLAVGVDNIFILVQAYQRDERLQGET
Chimpanzee        GILIVLSSVACSLGVFSYIGLPLTLIVIEVIPFLVLAVGVDNIFILVQAYQRDERLQGET
Gorilla           GILIVLSSVACSLGVFSYIGLPLTLIVIEVIPFLVLAVGVDNIFILVQAYQDDERLQGER
Marmoset          GILIVLSSVSCSLGVFSYIGFPLTLIVIEVIPFLVLAVGVDNIFILVQAYQRDERLQGET
Macaque           GILIVLSSVACSLGIFSYIGLPLTLIVIEVIPFLVLAVGVDNIFILVQAYQRDERLQGET
Gibbon            GILIVLSSVACSLGVFSYIGLPLTLIVIEVIPFLVLAVGVDNIFILVQAYQRDERLQGET
Baboon            GILIVLSSVGCSLGIFSYIGLPLTLIVIEVIPFLVLAVGVDNIFILVQAYQRDERLQGET
Bushbaby          GILIVLSSVTCSLGIFSYIGIPLTLIVIEVIPFLVLAVGVDNIFILVQTYQRDERLQGET
Tarsier           GILIVLSSVACSLGIFSYVGLPLTLIVIEVIPFLVLAVGVDNIFILVQTYQRDEHLQGET
Mouse             GILIVLSSVACSLGIFSYMGMPLTLIVIEVIPFLVLAVGVDNIFILVQTYQRDERLQEET
Rat               GILIVLSSVTCSLGIFSYIGMPLTLIVIEVIPFLVLAVGVDNIFILVQTYQRDERLQEET
Hamster           GILIVLSSVACSLGVFSYMGMPLTLIVIEVIPFLVLAVGVDNIFILVQTYQRDERLQEET
Guinea pig        GILIVLSSVSCSLGIFSYIGIPLTLIVIEVIPFLVLAVGVDNIFILVQTYQRDERLQGET
Opossum           GILMVLSSVVCSLGLFSYIGIPLTLIVIEVIPFLVLAVGVDNIFILVQTFQRDERLQGET
Tasmanian devil   GILMVLSSVVCSLGLFSYIGIPLTLIVIEVIPFLVLAVGVDNIFILVQTFQRDERLQGET
Platypus          GILIVLSSVACSLGIFSYAGIPLTLIVIEVIPFLVLAVGVDNIFILVQTFQRDQRLQGET
Elephant          GILIVLSSVACSLGIFSYVGIPLTLIVIEVIPFLVLAVGVDNIFILVQTYQRDERHQGET
Wallaby           GILMVLSSVVCSLGFFSYIGIPLTLIVIEVIPFLVLAVGVDNIFILVQTFQRDERLPGET
Sloth             GILIVLSSVVCSLGIFSYLGLPLTLIVIEVIPFLVLAVGVDNIFILVQAYQXXXXXXXXX
Hedgehog          GILIVLSSVACSLGIFSYLGIPLTLIVIEVIPFLVLAVGVDNIFILVQTYQRDERLQGET
Squirrel          GIVIVLSSVACSLGIFSYMGTPLTLIVIEVIPFLVLAVGVDNIFILVQTYQRDERLQGET


Tenrec            LDQHVGRVLGEVAPSMFLSSFSETVAFFLGALSVMPAVHTFSLFAGMAVFIDFLLQITCF
Macrobat          LDQQLGRILGEVA-SMLLSSFSETVAFFLGALSMMPAVYTFSLFAGMAVLIDFLLQITCF
Hyrax             LDQQVGRVLGEVAPSIFLSSFSETIAFFLGGLSTMPAVHTFSLFAGMAVFIDFLLQITCF
Tree shrew        LDQQLGRVLGEVAPSMFLSSFSETVAFFLGALSMMPAVHTFSLFAGMAVLIDFILQMTCF
Armadillo         XXXXXXXXXXXXXXXXXXXXXXXXXXXXALSVMPAVRTFSLFAGMAVFIDFLLQITCF
Kangaroo rat      LDQQLGRVLGEVAPTMFLSSFCETSAFFLGALSTMPAVHTFSLFAGMAILIDFLLQITCF
```

```
Shrew              LDQQLGRVLGEVAPSMFLSSFSETVAFFLGALSAMPAVHTFSLFAGMAVFIDFLLQISCF
Dolphin            LDQQVGRVLGEVAPSIFLSSFSETVAFFLGGLSVMPAVHTFSLFAGMAVLIDFLLQITCF
Cow                LDQQVGRVLGEVAPSMFLSSFAETVAFFLGGLSVMPAVHTFSLFAGMAVLIDFLLQITCF
Pig                LDQQLGRVLGEVAPSMFLSSFSETVAFFLGGLSVVPAVHTFSLFAGMAVLIDFLLQITCF
Weasel             LDQQLGRVLGEVAPSMFLSSFSEAVAFFLGALSKMPAVHTFSLFAGMAVLIDFLLQITCF
Panda              LDQQLGRVLGEVAPSMFLSSFSEAVAFFLGALSKMPAVHTFSLFAGMAVLIDFLLQITCF
Dog                LEQQLGRVLGEVAPSMFLSSFSEAVAFFLGALSQMPAVHTFSLFAGMAVLIDFLLQITCF
Cat                LDQQLGRVLGEVAPSMFLSSFSEAVAFFLGALSKMPAVHTFSLFAGMAVLIDFLLQITCF
Microbat           LDQQLGRVLGEVAPSMFLSSFSETVAFFLGALSVMPAVHTFSLFAGMAVLIDFLLQITCF
Horse              LDQQLGRVLGEVAPSMFLSSFSETVAFFLGALSVMPAVHTFSLFAGLAVLIDFLLQITCF
Mouse lemur        LDQQLGRVLGEVAPSMFLSSFSETIAFFLGALSMMPAVHTFSLFAGLAVFIDFLLQITCF
Pika               LDQQLGRVLGEVAPSIFLSAFAETVAFFLGGLSTMPAVHTFSLFAGMAVFIDFLLQITCF
Rabbit             LDQQLGRVLGEVAPSIFLSSFAETVAFFLGGLSVMPAVHTFSLFAGMAVFIDFLLQITCF
Orangutan          LDQQLGRVLGEVAPSMFLSSFSETVAFFLGALSMMPAVHTFSLFAGLAVFIDFLLQITCF
Human              LDQQLGRVLGEVAPSMFLSSFSETVAFFLGALSVMPAVHTFSLFAGLAVFIDFLLQITCF
Chimpanzee         LDQQLGRVLGEVAPSMFLSSFSETVAFFLGALSVMPAVHTFSLFAGLAVFIDFLLQITCF
Gorilla            EXXXXXXXXXXXXXXXXXXXXXXXXXXXXXXXXXPAVHTFSLFAGLAVFIDFILQITCF
Marmoset           LDQQLGRVLGEVAPSMFLSSFSETVAFFLGALSVMPAVHTFSLFAGLAVFIDFLLQITCF
Macaque            LDQQLGRVLGEVAPSMFLSSFSETVAFFLGAMSVMPAVHTFSLFAGLAVFIDFLLQITCF
Gibbon             LDQQLGRVLGEVAPSMFLSSFSETIAFFLGALSVMPAVHTFSLFAGLAVFIDFLLQITCF
Baboon             LDQQLGRVLGKVAPSMFLSSFSETVAFFLGAMSVMPAVHTFSLFAGLAVFIDFLLQITCF
Bushbaby           LDQQLGRVLGEVAPSMFLSSFSETIAFFLXXXXXXXXXXXXXXXXXXXXXXXXXXXXXXX
Tarsier            LDQQLGRVLGEVAPSMFLSSFSETVAFFLXXXXXXXXXXXXXXXXXXXXXXXXXXXXXXX
Mouse              LDQQLGRILGEVAPTMFLSSFSETSAFFFGALSSMPAVHTFSLFAGMAVLIDFLLQITCF
Rat                LDQQLGRILGEVAPTMFLSSFSETSAFFFGALSSMPAVHTFSLFAGMAVLIDFLLQITCF
Hamster            LDQQLGRILGEVAPTMFLSSFSETSAFFFGALSSMPAVHTFSLFAGLAVLIDFLLQITCF
Guinea pig         LDQQLGRVLGEVAPSMFLSSFCETTAFFLGALSAMPAVHTFSLFAGLAVFIDFLLQMTCF
Opossum            LDKQLGRILGEVAPSMLLSSFSEAIAFFLGALSTMPAVRTFSLFAGMAVFIDFLLQITCF
Tasmanian devil    LDKQLGRILGEVAPSMFLSSFSETIAFFLGALSTMPAVRTFSLFAGMAVFIDFLLQITCF
Platypus           LDAQMGRILGEVAPSMFLSSFSETVAFFLGGLSTMPAVHTFSLFAGMAVFIDFLLQITFF
Elephant           LDQQVGRVLGEVAPSMFLSSFSETAAFFLGGLSVMPAVHTFSLFAGMAVFIDFLLQMTCF
Wallaby            LDKQLGRILGEVAPSMFLSSFSETVAFFLGALSTMPAVRTFSLFAGMAVFIDFLLQITCF
Sloth              XXXXXXXXXXXXXXXXXXXXXXXXXXXXXGLSLMPAVHTFSLFAGMAVFIDFLLQITCF
Hedgehog           LDQQLGRVLGEVAPSMFLSSFSETVAFFLGALSVMPAVHTFSLFAGMAVFIDFLLQITCF
Squirrel           LDQQLGRVLGEVAPSMFLSSFCETSAFFFXXXXXXXXXXXXXXXXXXXXXXXXXXXXXXX


Tenrec             VSLLGLDIKRQEKSRLDILCCVSGPDDGTSIQASESFLFQFFKQFYAPLLLKDWMRPIVV
Macrobat           VSLLGLDIKRQEKNRLDVLCCVRGAEDGTGIQASESCLFRFFKNSYSPLLLKDWMRPIVI
Hyrax              VSLLGLDIKRQEKNQLDILCCVRGADDGTRVQASESYLFRFFKNSYSPLLLKDWMRPIVV
Tree shrew         VSLLGLDIKRQENNQLDILCCVRGAEDGTGVQASEGYLFSFFKNFYSPLLLKDWMRPIVI
Armadillo          VSLLGLDIKRQEKNRLDILCCVRGPDDGTSVQASENCLFQFFKNFYSPLLLKDWIRPIVV
Kangaroo rat       VSLLGLDIKRQEKNQLDILCCVRGAADGRSVQASDSFLFRFFKNSYSPFLLKDWMRPIVI
Shrew              VSLLGLDIKRQERGRLDILCCTSVADDGTGVQASESFLFRFFRKSYSPFLLKDWMRPIVI
Dolphin            VSLLGLDVKRQERNRLDVLCCVGGATDRAGIQASESCLFCFFRDAYAPFLLKDWMRPIVV
Cow                VSLLGLDIKRQEKNQLDVLCCVGGAADDAGIQASESCLFRFFRNSYAPLLLKDWMRPLVV
Pig                VSLLGLDIKRQEKNRLDVVCCVQGAEDGAGVQASESCLFRFFKNSYAPLLLKDWMRPIVI
Weasel             VSLLGLDIKRQEKHRLDVLCCVRGSEDGTSVQTSESCLFRFFKNSYSPLLLKDWMRPIVI
Panda              VSLLGLDIKRQENNRLDVVCCVRGSEDGTSVQASESCLFRFFKNSYSPLLLKEWMRPIVV
Dog                VSLLGLDLKRQEKNRLDVLCCLTGSEGGTGIQASESCLFRFFKNSYSPFLLKDWMRPIVI
Cat                VSLLGLDIKRQEKNRLDVLCCVRGSEDGTSVQASESCLFRLFKHSYSPLLLRDWMIPIVI
Microbat           VSLLGLDVKRQENNRLDILCCVRGGEDGAGLQASESCLFRFFRDSYSPLLLKAWMRPIVM
Horse              VSLLGLDIKRQEKNRLDVLCCLRGSEDGTSVQASESFLFRFFRNSYSPLLLKDWMRPIVI
Mouse lemur        VSLLGLDIKRQEKNRLDILCCVRGAEDGTGVQASESCLFRFFKHSYSPLLLKEWMRPIVI
Pika               VSLLGLDIKRQERNQLDICCCFRGAEAGTSIQASESYLFRFFKNSYSPLLLKDWMRPIVI
Rabbit             VSLLGLDIKRQEKNQLDVLCCFRGAEAGTSIQASESYLFRFFKNSYSPLLLKDWMRPIVI
Orangutan          VSLLGLDIKRQEKNRLDIFCCVRGAEDGTSVQASESYLFRFFKNSYSPLLLKDWMRPIVI
Human              VSLLGLDIKRQEKNRLDIFCCVRGAEDGTSVQASESCLFRFFKNSYSPLLLKDWMRPIVI
Chimpanzee         VSLLGLDIKRQEKNRLDIFCCVRGAEDGTSVQASESCLFRFFKNSYSPLLLKDWMRPIVI
Gorilla            VSLLGLDIKRQEKNRLDIFCCVRGAEDGTSVQASESCLFRFFKNSYSPLLLKDWMRPIVI
Marmoset           VSLLGLDIKRQEKNRLDIFCCIKGVEDGTSVQASESCLFRFFKNSYAPLLLKDWMRPIVV
Macaque            VSLLGLDINRQEKNRLDIFCCVRGAEDGTSVQASESCLFRFFKNSYSPLLLKDWMRPIVI
Gibbon             VSLLGLDIKRQEKNRLDIFCCVRGAEDGTSVQASESCLFRFFKNSYSPLLLKDWMRPIVI
Baboon             VSLLGLDINRQEKNRLDIFCCVRGAEDGTSVQASESCLFRFFKNSYSPLLLKDWMRPIVI
Bushbaby           XXXXXXXXXXXXXXXXXXXXXXXXXXXXXXXXXXXXXXXXXXXXXXXXXXXXXXXXXXI
Tarsier            XXXXXXXXXXXXXXXXXXXXXXXXXXXXXXXXXXXXXXXXXXXXXXXXXXXXXXXXXXI
```

```
Mouse              VSLLGLDIKRQEKNHLDILCCVRGADDGQGSHASESYLFRFFKNYFAPLLLKDWLRPIVV
Rat                VSFLGLDIKRQEKNRLDILCCVRGPDDGQESQASESYLFRFFKNAFAPFLLTDWLRPVVM
Hamster            VSLLGLDIKRQEKNRLDILCCVGGTDNGRGIQASESYLFRFFKNSFAPFLLKDWLRPIVI
Guinea pig         VSLLGMDIRRQEKNRLDILCCVQGANDGRSVQASESCLFHFFKNSYSPLLLKDWMRPLVV
Opossum            VSLLGLDIKRQEKNKLDILCCVKIAEDRTGPQPSESYLFKFFKNVFSSILLKDWMRPIVI
Tasmanian devil    VSLLGLDIKRQEKNKLDILCCVRIAEDRTDSQPSESYLFQFFKNAFSPFLLKDWMRPIVI
Platypus           VSLLGLDVKRQERNRLDILCCVRGGDEAAGGQPSQGYLFQFFKNVYSPLLLKDWMRPLII
Elephant           VSLLGLDIKRQEKNRLDILCCVRGADDGASVQASESCLFRFFKNSYSPLLLKDWMRPIVV
Wallaby            VSLLGLDIKRQEKNKLDILCCVRIAEDRTSPQPSESCLFQFFKNGFSPLLLKDWMRPLVI
Sloth              VSLLGLDIKRQERNRLDILCCVRGTDDGTSIQASENCLFQFFKNSYSPLLLKDWMRPIVV
Hedgehog           VSLLGLDIKRQEKNRMDILCCVRGAEDGTSVQASESYLFRFFKNSYSPFLLKDWMRPIVV
Squirrel           XXXXXXXXXXXXXXXXXXXXXXXXXXXXXXXXXXXXXXXXXXXXXXXXXXXXXXXXXXXI

                                  858
Tenrec             AVFVGFLSFSIAVLNKVEIGLDQSLSMPSDSYVKDYFQSLSQYLHAGPPVYFVLEEGHDY
Macrobat           AVFMGVLSFSIAVLNKVEIGLDQSLSVPDDSYVMDYFKSLSQYLHAGPPVYFVLEEGHNY
Hyrax              AIFVGVLSFSIAVLNKVEIGLNQSLSMPDDSYVIDYLKSLSQYLHAGPPVYFVLEEGHXX
Tree shrew         AMFVGVLSFSIAVLNKVEIGLDQSLSMPDDSYVVDYFKSLSQYLHAGPPVYFVLEEGHDY
Armadillo          AVFVGVLSFNIAVLNKVEIGLDQARSMPDXXXXXXXXXXXXXXXXXXXXXXXXXXXXXXX
Kangaroo rat       AVFVGILSFSIAVLNKVEIGLDQSLSMPNDSYVTDYFRSLKQYLHSGPPVYFVLEEGHDY
Shrew              AVFVGVLSFSIAVLNKVEIGLDQALSMPDDSYVVDYFKSLSQYLHAGPPVYFVVQEGHNY
Dolphin            AVFVGVLSFSIAVLNKVEIGLDQSLSMPDDSYVTDYFQSLSRYLHAGPPVYFVMEGGHDY
Cow                AVFVGVLSFSIAVLNKVEIGLDQSLSMPDDSYVTDYFQSLNQYLHAGPPVYFVLEEGHDY
Pig                AVFVGVLSFSIAVLNKVEIGLDQSLSMPDDSYVMDYFQSLSRYLHAGPPVYFVVEEGHNY
Weasel             AVFVGVLSFSIAVLNKVEIGLDQSLSMPDDSYVLDYFTSLR-YLHAGPPVYFVLEEGHDY
Panda              AVFVGVLSFSIAVLNKVEIGLDQSLSMPDDSYMLDYFRSLK-SLHAGPPVYFVLEEGHDY
Dog                AVFVGILSFSIAVLNKVEIGLDQSLSMPDDSYMMDYFKSLK-YLHAGPPVYFVLEEGHDY
Cat                AIFVGVLSFSVAVLNKVEIGLDQSLSMPDXXXXXXXXXXXX-XXXXXXXXXXXXXXXXXX
Microbat           AVFVGVLSFSIAVLNKVEIGLDQSLSMPEDSYVMDYFRSLSRFLHAGPPVYFVVEEGLDY
Horse              AVFVGVLSFSIAVLNKVEIGLDQFLSMPDDSYVIDYFQSIRQYLHAGPPVYFVLEEGHDY
Mouse lemur        AVFVGVLSFSIAVLNKVEIGLDQHLSMPDDSYVVDYLKSISQYLHAGPPVYFVVEEGHDY
Pika               AVFVGILSFSIAVLNKVEIGLDQSLSMPDDSYVVDYFRSLGQYLHAGPPVYFVLEEGHDY
Rabbit             AVFVGVLSFSIAVLNKVEIGLDQSLSMPDDSYVVDYFKSLGQYLHAGPPVYFVLEEGHNY
Orangutan          AIFVGVLSFSIAVLNKVDIGLDQSLSMPDDSYVVDYFKSISQYLHAGPPVYFVLEEGHDY
Human              AIFVGVLSFSIAVLNKVDIGLDQSLSMPDDSYMVDYFKSISQYLHAGPPVYFVLEEGHDY
Chimpanzee         AVFVGVLSFSIAVLNKVDIGLDQSLSMPDDSYVVDYFKSISQYLHAGPPVYFVLEEGHDY
Gorilla            AIFVGVLSFSIAVLNKVDIGLDQSLSMPDDSYVVDYFKSISQYLHAGPPVYFVLEEGHDY
Marmoset           AVFVGVLSFSIAVLNKVDIGLDQSLSMPDDSYVVDYFKSISQYLHAGPPVYFVLEEGHDY
Macaque            AIFVGVLSFSIAVLNKVDIGLDQFLSMPDDSYVVDYFKSMSQYLHAGPPVYFVLEEGHDY
Gibbon             AIFVGVLSFSIAVLNKVDIGLEQSLSMPDDSYVVDYFKSISQYLHAGPPVYFVLEEGHNY
Baboon             AIFVGVLSFSIAVLNKVDIGLDQFLSMPDDSYVVDYFKSMSQYLHAGPPVYFVLEEGHDY
Bushbaby           AVFVGVLSFSVAVLNKVEIGLDQYLSMPDDSYVVDYFKSISQYLHAGPPVYFVLEEGHNY
Tarsier            AVFVGVLSFSIAVLNKVEIGLDQSLSMPDDSYVVDYFKAISQYLHAGPPVYFVLEEGHDY
Mouse              AVFVGVLSFSVAVVNKVDIGLDQSLSMPNDSYVIDYFKSLAQYLHSGPPVYFVLEEGYNY
Rat                AVFVGILSFSIAVVNKVEIGLDQSLSMPNDSYVIDYFKSLGQYLHSGPPVYFVLEEGYNY
Hamster            AVFVGVLSFSIAVMNKVEIGLDQSLSMPNDSYVIDYFKSLGQYLHSGPPVYFVLEEGHDY
Guinea pig         AIFVGLLSFSVAVLNKVEIGLDQSLSMPDDSYVIDYFRSLGQYLHAGPPVYFVVEEGHDY
Opossum            SVFVGILSFSIAVLNKVEIGLDQSLSMPDDSYVLDYFKSLNQYLHAGPPVYFVLEEGHDY
Tasmanian devil    SVFVGILSFSIAVLNKVEIGLDQSLSMPDDSYVLDYFKSLNQYLHAGPPVYFVLEEGHNY
Platypus           SVFVGILSFSIAVLNKVEIGLDQSLSMPDDSYVMDYFKALGQYLHAGPPVYFVLEEGHDY
Elephant           AIFVGVLSFSIAVLNKVEIGLSQSLSMPDDSYVMDYFKSLSQYLHAGPPVYFVLEEGHDY
Wallaby            SVFVGILSFSIAVLNKVEIGLDQSLSVPDXXXXXXXXXXXXXXXXXXXXXXXXXXXXXXX
Sloth              TVFVGVLSFSIAVLNKVEIGLDQALSMPDDSYVVDYFQSLSRYLHAGPPVYFVLEEGHDY
Hedgehog           AVFVGVLSFSIAVMNKVEIGLDQSLSMPDDSYMTDYFKFLGQYLHAGPPVYFVLEEGHDY
Squirrel           AVFVGVLSFSIAVLNKVEIGLDQSLSMPDDSYVIDYFKSLGQYLHSGPPVYFVLEEGHNY

                                                    931
Tenrec             TSLKGQNMVCGGTGCSNDSLVQQIFNAAQLDSYTRIGFAPSSWIDDYFDWIKPQSSCCRV
Macrobat           TSLAGQNMVCGGVGCNSDSLVQQIFNAAELENYTRIGFAPSSWIDDYFDWIKPQSSCCRV
Hyrax              XXXXXXXXXXXXXXXXXXXXXXXXXXXXXXXXXXXXXXXXXXXXXXXXXXXXXXXXXXXX
Tree shrew         TSLRGQNMVCGGTGCDNNSLVQQLFDAAQLDSYTRIGFAPSSWIDDYFDWVKPQSSCCRI
Armadillo          XXXXXXXXXXXXXXXXXXXXXXXXXXXXXXXXXXXXXXXXXXXXXXXXXXXXXXXXXXXX
Kangaroo rat       TSQEGQNMVCGGMGCDNDSLVQQIFNAAQLDNYTRIGFAPSSWIDDYFDWVKPQSSCCRV
Shrew              TSLKGQNMVCGGMGCDNDSLVQQIFNAAQMDNYTRIGFAPSSWIDDYFDWIKPQSSCCRV
Dolphin            ASLKGQNMVCGGLGCNNDSLVQQIFTAAQLDNYTRIGFALSSLNDDYFDWVKPQSSCCRV
Cow                TSTKGQNMVCGGLGCNNDSLVQQVFTAAQLDSYTRIGFAPSSWIDDYFDWVKPQSSCCRI
```

```
Pig               TSLKGQNMVCGGLGCNNDSLVQQIFTAAQLDNYTRIGFAPSSWIDDYFDWIKPQSSCCRV
Weasel            SSLKGQNMVCGGMGCNNDSLVQQIFTAAQLDNYTRIGFAPSSWIDDYFDWVKPQSSCCRV
Panda             SSLKGQNMVCGGTGCNNDSLVQQIFTAAQLNNYTRIGFAPSSWLDDYFDWVKPQSSCCRV
Dog               TSLEGQNMVCGGMGCNNDSLVQQIFSAAQLDNYTRIGFAPSSWIDDYFDWVKPQSSCCRV
Cat               XXXXXXXXXXXXXXXXXXXXXXXXXXXXXXXXTRIGFAPSSWIDDYFDWVKPQSSCCRV
Microbat          TSLQGQNLVCGGMGCNNDSLVQQLFDAAELDAYTRIGFAPSSWIDDYFDWVKPQSSCCRV
Horse             TSLKGQNMVCGGMGCDNDSLVQQIFTAAQLDSYTRIGFAPSSWIDDYFDWVKPQSSCCRV
Mouse lemur       TSPKGQNMVCGGVGCNNDSLVQQIFNAAQLDNYTRIGFAPSSWIDDYFDWVKPLSTCCRV
Pika              TSLQGQNMVCGGMGCDNDSVVQQIFNAAQLDNYTRIGFAPSSWIDDYFDWVKPQSSCCRV
Rabbit            TSLQGQNMVCGGLGCDNDSLVQQIFNAAQLDNYTRIGFAPSSWIDDYFDWVKPQSSCCRV
Orangutan         TSPKGQNMVCGGMGCNNDSLVQQIFNAAQMDNYTRIGFAPSSWIDDYFDWVKPQSSCCRV
Human             TSSKGQNMVCGGMGCNNDSLVQQIFNAAQLDNYTRIGFAPSSWIDDYFDWVKPQSSCCRV
Chimpanzee        TSSKGQNMVCGGMGCNNDSLVQQIFNAAQLDNYTRIGFAPSSWIDDYFDWVKPQSSCCRV
Gorilla           TSSKGQNMVCGGMGCNNDSLVQQIFNAAQLDNYTRIGFAPSSWIDDYFDWVKPQSSCCRV
Marmoset          TSPKGQNMVCGGMGCNNDSLVQQIFNAAQLDNYTRIGFAPSSWIDDYFDWVKPQSSCCRV
Macaque           TSPKGQNMVCGGMGCNNDSLVQQIFNAAQLDNYTRIGFAPSSWIDDYFDWVKPQSSCCRV
Gibbon            TSPKGQNMVCGGTGCNNDSLVQQIFNAAQLDNYTRIGFAPSSWIDDYFDWVKPQSSCCRV
Baboon            TSPKGQNMVCGGMGCNNDSLVQQIFNAAQLDNYTRIGFAPSSWIDDYFDWVKPQSSCCRV
Bushbaby          TSLQGQNMVCGGMGCNNDSLVQQIFNAAQLDNYTRIGFAPSSWIDDYFDWVKPQSSCCRV
Tarsier           TSPTGQNMVCGGMGCNNDSLVQQIFNAAQLDNYTRIGFAPSSWLDDYFDWVKPQSSCCRV
Mouse             SSRKGQNMVCGGMGCDNDSLVQQIFNAAELDTYTRVGFAPSSWIDDYFDWVSPQSSCCRL
Rat               SSRRGQNMVCGGMGCDNDSLVQQIFNAAELDTYTRVGFAPSSWIDDYFDWVSPQSSCCRL
Hamster           TTHKGQNMVCGGMGCDNDSLVQQIFNAAELDNYTRIGFAPSSWIDDYFDWVAPQSSCCRL
Guinea pig        TSRPGQNMVCGGMGCDNDSLVQQIFNAAQLDNYTRIGFAPSSWIDDYFDWVKPQSSCCRL
Opossum           TSLEGQNMVCGGMGCNNNSLVQQIFNAAELDNYTRIGFAPSSWIDDYFDWIKPQSSCCRI
Tasmanian devil   TSLEGQNMVCGGMGCNNNSLVQQIFNAAELENYTKIGFAPSSWIDDYFDWIKPQSSCCRI
Platypus          TTLEGQNMVCGGMGCNNNSLVQQIFNAAELDNYTRIGFAPSSWIDDYFDWVKPQSSCCRV
Elephant          TSLRGQNMVCGGMGCDNDSLVQQLFNAAELDSYTRVGFAPSSWIDDYFDWVKPQSSCCRV
Wallaby           XXXXXXXXXXXXXXXXXXXXXXXXXXXXXXXXXXXXXXXXXXXXXXXXXXXXXXXXXXXX
Sloth             TSLAGQNVVCGGTGCNNDSLVQQIFNAAELDSYTRIGFTPSSWIDDYFDWIKPQSSCCRV
Hedgehog          TSLKGQNMVCGGMGCNNDSLVQQIFTAAQLDNYTRIGFTPXXXXXXXXXXXXXXXXXXXX
Squirrel          ASLQGQNMVCGGMGCDNDSLVQQIFNAAQLDNYTRIGFAPSSWIDDYFDWVKPQSTCCRV


Tenrec            FNVTEQFCNASVVDPACVRCRPLTPEGKQRPRGKDFMKFLPMYLSDNPNPKCGKGGHAAY
Macrobat          YNNTDQFCNASVADPTCIRCRPLTPEGKQRPQGGDFMRFLPMFLSDNPNPKCGKGGHAAY
Hyrax             XXXXXXXXXXXXXXXXXXXXXXXXXXXXXXXXXXXXXXXXXXXXXXXXXXXXXXXGHAAY
Tree shrew        YNITDQFCNASVVDPACVRCRPLTPEGKQRPQGGDFMRFLPMFLSDNPNPKCGKGXXXXX
Armadillo         XXXXXXXXXXXXVDPACVRCRPLTPEGKQRPQGEDFMKFLPLFLSDNPNPKCGKGGHAAY
Kangaroo rat      YNSTDQFCNASVVNPECIRCRPLTPENKQRPQNRDFMKFLPMFLSDTP--KCGKGGHAAY
Shrew             YNSTDQFCNASVVDPTCLRCRPLTPEGKQRPQGGDFMRFLPMFLSDNPNPKCGKGGHAAY
Dolphin           YNSTDRFCNASVVDPACVHCRPLTPEGKQRPQGADFMRFLPMFLSDNPNPKCGKGGHAAY
Cow               YNSTEQFCNASVVNPTCVRCRPLTPEGKQRPQGADFMRFLPMFLSDNPNPKCGKGGHAAY
Pig               YNSTDQFCNASVVDPTCIRCRPLTSEGKQRPQGEDFMRFLPMFLSDNPNPKCGKGGHAAY
Weasel            YNGTDRFCNASVVDPACGRCRPLTPEGKQRPQGEDFMRFLPMFLSDNPNPKCGKGGHAAY
Panda             YNSTDTFCNASVVDPGCVRCRPLTPEGKQRPQGGDFMRFLPMFLSDNPNPKCGKGGHAAY
Dog               YNSTDQFCNASVVDPACVRCRPLTQEGKRRPQGEDFMRFLPMFLSDNPNPKCGKGGHAAY
Cat               YNSTDRFCNASVVDPACIRCRPLTQEGKQRPQGGDFMRFLPMFLSDNPNPKCGKGGHAAY
Microbat          HNGSGQFCNASVADPGCIRCRPLTPEGKQRPQGGDFMRFLPMFLSDNPNPKCGKGGHAAY
Horse             YNSTDQFCNASVVDPACVRCRPLTPEGKQRPQGRDFMRFLPMFLSDNPNPRCGKGGHAAY
Mouse lemur       YNTSGQFCNASVADPACVRCRPLTPEGKQRPQGGDFMRFLPMFLSDNPNPKCGKGGHAAY
Pika              SNVTEQFCNASVVDPACVRCRPLTPEGKQRPQGEDFMRFLPMFLSDNPNPKCGKGGHAAY
Rabbit            SNVTEQFCNASVVDPACVRCRPLTPEGKQRPQGEDFMRFLPMFLSDNPNPKCGKGGHAAY
Orangutan         DNITDQFCNASVVDPACVRCRPLTPEGKQRPQGGDFMRFLPMFLSDNPNPKCGKGGHAAY
Human             DNITDQFCNASVVDPACVRCRPLTPEGKQRPQGGDFMRFLPMFLSDNPNPKCGKGGHAAY
Chimpanzee        DNITDQFCNASVVDPACVRCRPLTPEGKQRPQGGDFMRFLPMFLSDNPNPKCGKGGHAAY
Gorilla           DSITDQFCNASVVDPACVRCRPLTPEGKQRPQGGDFMRFLPMFLSDNPNPKCGKGGHAAY
Marmoset          DNITDQFCNASVVDPACIRCRPLTAEGKQRPQGRDFMKFLPMFLSDNPNPKCGKGGHAAY
Macaque           DNITDQFCNASVVDPACVRCRPLTPEGKQRPQGGDFMRFLPMFLSDNPNPKCGKGGHAAY
Gibbon            DNITDQFCNASVVDPACVRCRPLTPEGKQRPQGGDFMRFLPMFLSDNPNPKCGKGGHAAY
Baboon            DNITDQFCNASVVDPACVRCRPLTPEGKQRPHGGDFMRFLPMFLSDNPNPKCGKGGHAAY
Bushbaby          YNTTEQFCNASVVDPACVRCRPLTPEGKQRPQGEDFMRFLPMFLADNPNPKCGKGGHAAY
Tarsier           YNITEQFCNASVVDPTCVRCRPLTPEGKQRPQGGDFMRFLPMFLSDNPNPKCGKGGHAXX
Mouse             YNVTHQFCNASVMDPTCVRCRPLTPEGKQRPQGKEFMKFLPMFLSDNPNPKCGKGGHAAY
Rat               YNVTHQFCNASVIDPTCVRCRPLTPEGKQRPQGKEFMKFLPMFLSDNPNPKCGKGGHAAY
Hamster           YNATHQFCNASVIDPTCIRCRPLTPEGKQRPQGKEFMKFLPMFLSDNPNPKCGKGGHAAY
```

```
Guinea pig        YNATAQFCNASVIDPTCVRCRALTPEGKQRPQGGDFMRFLPMFLSDNPNPKCGKGGHAAY
Opossum           YNRTDKFCNASVVDPSCVRCRPLTPEGKRRPQGEDFMKFLPMFLSDNPNPKCGKGGHAAY
Tasmanian devil   YNMTERFCNASVVDPSCIHCRPLTPDGKRRPQGEDFMKFLPMFLSDNPNPKCGKGGHASY
Platypus          YNGTDLFCNASVVDPSCVRCRPLTPEGKQRPQGSDFLHFLPMFLSDNPNPKCGKGGHAAY
Elephant          SNITEQFCNASVVDPTCVRCRPLTPEGKQRPQGKDFMKFLPMFLSDNPNPKCGKGGHAAY
Wallaby           XXXXXXXXXXXXXXXXXXXXXXXXXXXXXXXXXXXXXXXXXXXXXXXXXXXXXXXGHASY
Sloth             YNTTEQFCNASXXXXXXXXXXXXXXXXXXXXXXXXXXXXXXXXXXXXXXXXXXXXGHAAY
Hedgehog          XXXXXXXXXXXXXXXXXXXXXXXXXXXXXXXXXXXXXRFLPMFLSDNPNPSCGKGGHAAY
Squirrel          YNITDQFCNASXXXXXXXXXXXXXXXXXXXXXXXXXXXXXXXXXXXXXXXXXXXXGHAAY


Tenrec            SSAVNILANNTRVGATYFMTYHTVLQTSADFIDAMKKARLLATNIMETMGINGSQYRLFP
Macrobat          GSAVNILGNDTSVGATYFMTYHTVLQTSADFIDAMRKARLIAGNITKTMSQEGSNHHVFP
Hyrax             GSAVNILGNNTSVGATYFMTYHTVLQTSADFIDALKKARLVANNITETMGINRSHYRVFP
Tree shrew        XXXXXXXXXXXXXXXXYFMTY-TVLQNSTDFIDAMKKARLIASNITETMGINGSNYRVFP
Armadillo         GSAVNILGNNTRVGATYFMTYHTVLQTSADFIDAMKKARLVASNITDTMGVRGXXXXXXX
Kangaroo rat      SSAVNILGNDTGVGATYFMTYHTVLQTSADFIDAMKKAQLVASNITKTMGMKGSNYRVFP
Shrew             GSAVNILGNDTGVGATYFMTYHTVLQRSADFIDAMSKARLIAANITRTMGLENSSYRVFP
Dolphin           SAAVNILGNGTGVGATYFMTYHTVLKTSADFIDAMKKARLIASNITRTMGLEGSDHRVFP
Cow               SAAVNILDNGTRVGATYFMTYHTVLQTSADFIDAMEKARLIASNITRTMNQQGGDHRVFP
Pig               SSAVNILGNGSGVGATYFMTYHTVLQASADFIDAMQKARLIASNITRTMGLEASSYRVFP
Weasel            SSAVNILGNDTAVGATYFMTYHTVLQTSADFTDAMRKAILIAGNITKTMGLEGSGYRVFP
Panda             SSAVNILGNNTAVGATYFMTYHTVLQTSADFTDAMRKAIVIASNITKTMGLEGSDYRVFP
Dog               GSAVNLVGNDTSVGATYFMTYHTVLQTSADFTDAMRKAILIASNITKTMSLKGSHYRVFP
Cat               SSAVNILGNDTGVGATYFMTYHTVLQTSADFTDAMRKANLIASNITKTMGLEGSNYRVFP
Microbat          GSAVNILGNDTGVGATYFMTYHTVLQTSADFIDAMRKARLIAGNITQTMSQGGSNYRVFP
Horse             SSAVNILGNDTGVGATYFMTYHTVLQTSADFIDAMKKARLIAGNITKTMGLEGGNYRVFP
Mouse lemur       SSAVNILGSDTGVGATYFMTYHTVLQTSADFIDAMKKARLIS-NITETMG--CSSYRVFP
Pika              ATAVNILGNNTAVGATYFMTYHTVLQNSADFIDAMKKAKLVASNITATMGLKGSHQRVFP
Rabbit            GTAVNILGNGTEVGATYFMTYHTVLQTSADFIDAMKKAQLIAGNATATMGLKGSRCRVFP
Orangutan         SSAVNILGNGTRVGATYFMTYHTVLQTSADFIDALKKARLVASNVTETMGINGSAYRVFP
Human             SSAVNILGHGTRVGATYFMTYHTVLQTSADFIDALKKARLIASNVTETMGINGSAYRVFP
Chimpanzee        SSAVNILGNGTRVGATYFMTYHTVLQTSADFIDALKKARLIASNVTETMGINGSAYRVFP
Gorilla           SSAVNILGNGTRVGATYFMTYHTVLQTSADFIDALKKARLIASNVTETMGINGSAYRVFP
Marmoset          SSAVNILSNGTGVGATYFMTYHTVLQTSADFIDALKKARLIASNITETMGINGSAYRVFP
Macaque           SSAVNILGNGTRVGATYFMTYHTVLQTSADFIDALKKARLIASNVTETMGINGSAYRVFP
Gibbon            SSAVNILGNGTRVGATYFMTYHTVLQTSADFIDALKKARLIASNVTETMGINGSAYRVFP
Baboon            SSAVNILGNGTRVGATYFMTYHTVLQTSADFIDALKKARLIASNVTETMGINGSAYRVFP
Bushbaby          SSAVNILSNDTGVGATYFMTYHTVLQTSADFIDAMKKARLIASNITETMGINGSGYRVFP
Tarsier           XXXXXXXXXXXXXXXXXXXXXXXXXXXXXXXXXXXXXXXXXXXXXXXXXXXXXXXXXXXX
Mouse             GSAVNIVGDDTYIGATYFMTYHTILKTSADYTDAMKKARLIASNITETMRSKGSDYRVFP
Rat               SSAVNIMGDDTYIGATYFMTYHTILKTSADYIDALKKARLIASNITETMRSKGSDYRVFP
Hamster           SSAVNIIGDDTYVGATYFMTYHTVLKTSADYIDAMKKAQLVARNITETMNSKGSNYRVFP
Guinea pig        GSAVNLLGNATGVGATYFMTYHTVLQTSTDYIDALRKARLVADNITRTMSAKGSNYRVFP
Opossum           SSAVHLTNNRSEVGATYFMTYHTVLHSSSDYIDALKKARMVAANITDTMGLAGRPYRVFA
Tasmanian devil   SAAVHFKNNYTEIGATYFMTYHTVLHTSSDYIDAMRKARMVAANITDTMGLQDKNYRVFP
Platypus          SSAVNLANGSTSIGATYFMTFHTVLKTSSDFIDAMQKARTVAQNITESVGLPEGGYRVFP
Elephant          GSAVNILGNNTSVGATYFMTSHTVLQTSADFIDAMKKARLIASNITETMGRKGSNYRVFP
Wallaby           SSAVHIKNNHTEVGATYFMTYHTVLHASSDYIDAMRKARIVASNITETMGLQNKNYRVFP
Sloth             GSAVNILGNSTAVGATYFMTYHTVLKTSADFIDAMKKARLVASNITDTMGIKEKNYRVFP
Hedgehog          GSAVNILGNDTSVGATYFMTYHTVLQTSADFIDAMKKARLVADNITKTMGLEESSYRVFP
Squirrel          SSAVNILGNNTGIGATYFMTYHTVLQTSADFIDAMKKARLVASNITETMSTKGSNYRVFP


Tenrec            YSVFYVFYEQYLTIIDDTIFNLGVSLGAIFLVTLVLLGCDLWSAVIMCVTIAMILVNMFG
Macrobat          YSVFYVFYEQYLTIIDDTIFNLCVSLGAIFLVTVVLLGCELWSAVIMCVTIAMILVDMFG
Hyrax             YSVFHVFYEQYLTIIDDTIFNLGVSLGAIFLVTFVLLGCELWSAVIMCVTIAMILVNMFG
Tree shrew        YXXXXXXXXXXXXXXXXXXXXXXXXXXXXXXXXXXXXXXXXXXXXXXXXXXAMIVVNMFG
Armadillo         XXXXXXXXXXXXXXXXXXXXXXXXXXXXXXXXXXXXXXXXXXXXXXXXXXXXXXXXXXXX
Kangaroo rat      YSVFYVFYEQYLTIIDDAIFNLSMSLGSIFLVTMVVLGCDLWSAVIMCVTIAMILVNMFG
Shrew             YSEFYVFYEQYLTIVVDTIFNLSVSPAAILLVTMVLLSCELRAAVIMCSSITMILXXXXX
Dolphin           YSVFYVFYEQYLTMIDDTLFNLGVSLGAIFVVTVVLLGCELWSAVIMCVTIAMILVNMFG
Cow               YSVFYVFYEQYLTMIDDTIFNLSVSLGAIFLVAVVLLGCELWSAVIMCATIAMILVNMFG
Pig               YSVFYVFYEQYLTVIDDTIFNLGVSLGAIFLVTVVLMGCELWATVIMCVTIAMILVNMFG
Weasel            YSVFYVFYEQYLTIIDDTIFNLSVSLGAIFLVTLVLLGCELWSAVIMCVTIAMILVNMFG
Panda             YSVFYVFYEQYLTIIDDTIFNLGVSLGAIFLVTLVLLGCELWSAVIMCVTIAMILVNMFG
```

```
Dog                 YSVFYVFYEQYLTIIDDTIFNLSVSLGAIFLVTLVLLGCELWSAVIMCVTIAMILVNMFG
Cat                 YSVFYVFYEQYLTIIDDTIFNLSVSLGAIFLVTVILLGCDLWSAVIMCITIAMILVNMFG
Microbat            YSVFYVFYEQYLTIIEDTVFNLCVSLGAIFLVTVVLLGCELWSAVIMCVTIAMILVNMFG
Horse               YSVFYVFYEQYLTIIDDTIFNLGVSLGAIFLVTMVLLGCELWSAVILCVTIAMILVNMFG
Mouse lemur         YSVFYVFYEQYLTIIDDTVFNLGVSLGAVFLVAMVLLGCELWSAVIMCATIAMILVNMFG
Pika                YSVFYVFYEQYLTILDDTIFNLGVSLGAVFAVAVVLLGCELWAAVIMCVTITMILVNMFG
Rabbit              YSVFYVFYEQYLTILDDAVFNLGVSLGAVFVVAVVLLGCELWSAVIMCVTITMILVNMFG
Orangutan           YSVFYVFYEQYLTIIDDTIFNLGVSLGAIFLVTMVLLGCELWSAVIMCATIAMVLVNMFG
Human               YSVFYVFYEQYLTIIDDTIFNLGVSLGAIFLVTMVLLGCELWSAVIMCATIAMVLVNMFG
Chimpanzee          YSVFYVFYEQYLTIIDDTIFNLGVSLGAIFLVTMVLLGCELWSAVIMCATIAMVLVNMFG
Gorilla             YSVFYVFYEQYLTIIDDTIFNLGVSLGAIFLVTMVLLGCELWSAVIMCTTIAMVLVNMFG
Marmoset            YSVFYVFYEQYLTIIDDTVFNLGASLGAIFLVTMVLLGCELWSTVIMCTTIAMVLVNMFG
Macaque             YSVFYVFYEQYLTIIDDTIFNLGVSLGAIFLVTMVLLGCELWSAVIMCTTIAMVLVNMFG
Gibbon              YSVFYVFYEQYLTIIDDTIFNLGVSLGAIFLVTMVLLGCELWSAVIMCATIAMVLVNMFG
Baboon              YRVFYVFYEQYLTIIDDTIFNLGVSLGAIFLVTMVLLGCELWSAVIMCATIAMVLVNMFG
Bushbaby            YSVFYVFYEQYLTIIDDTIFNLGVSLGAVFLVAVVLLGCELWSAVLMCATIAMVLVNMFG
Tarsier             XXVFYVFYEQYLTIINDTIFNLGTSLGAIFLVTMVLLGCELWSAVLMCATIAMILVNMFG
Mouse               YSVFYVFYEQYLTIIDDTIFNLSVSLGSIFLVTLVVLGCELWSAVIMCITIAMILVNMFG
Rat                 YSVFYVFYEQYLTIIDDAIFNLSVSLGSIFLVTLVALGCELWSAVIMCLTIAMILVNMFG
Hamster             YSVFYVFYEQYLTIIDDTIFNLSVSLGSIFLVTLVVLGCELWSAVIMCITIAMILVNMFG
Guinea pig          YSVFYVFYEQYLTIIEDTVFNLGVSLGSIFLVTMVVLGCELWSAVIMCATIAMILVNMFG
Opossum             YSVFYVFYEQYLTIVDDTIFNLGVSLGAIFLVTAVLLGCDLWSAVIMCVTIAMILVNMFG
Tasmanian devil     YSVFYVFYEQYLTIVDDTIFNLGVSLGAIFLVTTILLGCDLWSSVIMCVTIAMILVNMFG
Platypus            YSVFYVFYEQYLTIVGDTIFNLGVSLGAIFLVTAVLLGCEVWAAVVLCLTIAMILVNMFG
Elephant            YSVFYVFYEQYLTIIDDTIFNLGVSLGAVFLVTFVLLGCELWSAVIMCVTIAMILVNMFG
Wallaby             YSVFYVFYEQYLTIVDDTIFNLGVSLGAIFLVTTVLLGCDVWSAVIMCVTIAMILVNMFG
Sloth               YSVFYVFYEQYLTIVDDTVFNLGVSLGAIFLVTVVLLGCELWAAVLMCTTIAMVLANMFG
Hedgehog            YSVFYVFYEQYLTIIDDTIFNLGVSLGAIFLVTMVLLGCEIWSAVIMCVTIAMILVNMFG
Squirrel            YSVFYVFYEQYLTIIDDTIFNLGVSLGSIFLVTVIVLGCELWSAVIMCVTIAMILVNMFG


Tenrec              VMWLWGISLNAVSLVNLVMSCGISVEFCSHITRAFTVSTKGSRVGRAEAALSCMGSSVFS
Macrobat            IMWLWGISLNAVSLVNLVMSCGISVEFCSHITRAFTVSAKGSRVERAEEALSHMGSSVFS
Hyrax               VMWLWGISLNAVSLVNLVMSCGISVEFCSHITRAFTVSTKSSRVGRAEEALSHMGSSVFS
Tree shrew          VMWLWGISLNAVSLVNLVMSCGISVEFCSHITRAFAVSTKGSRVARAEEALAHMGSSXXX
Armadillo           XXXXXXXXXXXXXXXXXXXXXXXXXXXXXXXXXXXXXXXXXXXXXXXXXXXXXXXXXVFS
Kangaroo rat        VMWLWGISLNAVSLVNLVMSCGXXXXXXXXXXXXXXXXXXXXXXXXXXXXXXXXXXXXXX
Shrew               XXXXXXXXXXXXXXXXXXXSCGISVEFCSHITRAFTVSARGSRVERAEEALSHMGSSVFS
Dolphin             VMWLWGISLNAVSLVNLVMSCGISVEFCSHITRAFTVSTKGSRVERAEEALSRMGSSVFS
Cow                 VMWLWGISLNAVSLVNLVMSCGISVEFCSHITRAFTVSTKGSRVERAEEALSHMGSSVFS
Pig                 VMWLWGISLNAVSLVNLVMSCGISVEFCSHITRAFTLSTKGSRVDRAEEALAHMGSSVFS
Weasel              VMWLWGISLNAVSLVNLVMSCGISVEFCSHITRAFTVSTKGSRVERAEEALSHMGSSVFS
Panda               VMWLWGISLNAVSLVNLVMSCGISVEFCSHITRAFTVSTKGSRVQRAEEALSHMGSSVFS
Dog                 VMWLWGISLNAVSLVNLVMSCGISVEFCSHITRAFTVSAKGSRVERAEEALSHMGSSVFS
Cat                 VMWLWGISLNAVSLVNLVMSCGISVEFCSHITRAFTVSMKGSRAQRAEEALAHMGSSVFS
Microbat            VMWLWGISLNAVSLVNLVMSCGISVEFCSHITRAFTVSAKGSRVARAEEALSHMGSSVFS
Horse               VMWLWGISLNAVSLVNLVMSCGISVEFCSHITRAFTVSAKGSRVERAEEALSHMGSSVFS
Mouse lemur         VMWLWGISLNAVSLVNLVMSCGISVEFCSHITRAFTVSTKGSRVARAEEALAHMGSSVFS
Pika                VMWLWGISLNAVSLVNLVMSCGISVEFCSHITRAFTVSGKGSRAARAEDALAHMGSSVFS
Rabbit              VMWLWGISLNAVSLVNLVMSCGISVEFCSHITRAFTVSGKGSRVARAEEALAHMGSSVFS
Orangutan           VMWLWGISLNAVSLVNLVMXXXXXXXXXXXXXXXXXXXXXXXXXXXXXXXXXXMGSSVFS
Human               VMWLWGISLNAVSLVNLVMSCGISVEFCSHITRAFTVSMKGSRVERAEEALAHMGSSVFS
Chimpanzee          VMWLWGISLNAVSLVNLVMSCGISVEFCSHITRAFTVSTKGSRVERAEEALAHMGSSVFS
Gorilla             VMWLWGISLNAVSLVNLVMSCGISVEFCSHITRAFTVSTKGSRVERAEEALAHMGSSVFS
Marmoset            VMWLWGISLNAVSLVNLVMSCGISVEFCSHITRAFTVSTKGSRVDRAEEALAHMGSSVFS
Macaque             VMWLWGISLNAVSLVNLVMSCGISVEFCSHITRAFTVSTKGSRVERAEEALAHMGSSVFS
Gibbon              VMWLWGISLNAVSLVNLVMSCGISVEFCSHITRAFTVSTKGSRVERAEEALAHMGSSVFS
Baboon              VMWLWGISLNAVSLVNLVMSCGISVEFCSHITRAFTVSTKGSRVERAEEALAHMGSSVFS
Bushbaby            VMWLWGISLNAVSLVNLVMSCGISVEFCSHITRAFTVSTKGSRVARAEEALAHMGSSVFS
Tarsier             VMWLWGISLNAVSLVNLVMSCGISVEFCSHITRAFTVSTKGSRVERAEQALAHMGSSXXX
Mouse               VMWLWGISLNAVSLVNLVMSCGISVEFCSHITRAFTMSTKGSRVSRAEEALAHMGSSVFS
Rat                 VMWLWGISLNAVSLVNLVMTCGISVEFCSHITRAFTMSTKGSRVSRAEEALAHMGSSVFS
Hamster             VMWLWDISLNAVSLVNLVMSCGISVEFCSHITRAFTMSTKGSRVSRAEEALAHMGSSVFS
Guinea pig          VMWLWGISLNAVSLVNLVMSCGISVEFCSHITRAFTVSCRKSRVDRAQEALAHMGSSIFS
Opossum             VMWLWGISLNAVSLVNLVMSCGISVEFCSHITRAFAVSTKGSRVARANEALSNMGSCVFS
Tasmanian devil     VMWLWGISLNAVSLVNLVMSCGISVEFCSHITRAFTMSTKGSRVARAEEALSHMGSCVFS
```

```
Platypus          VLWLWGISLNAVSLVNLVMSCGISVEFCSHITRAFTLSTRGSRVERAEEALANMGSSVFS
Elephant          VMWLWGISLNAVSLVNLVMSCGISVEFCSHITRAFTVSTKGSRVSRAEEALSHMGSSVFS
Wallaby           VMWLWGISLNAVSLVNLVMSCGISVEFCSHITRAFAVSRKRTRAARAEEALSHMGSCVFS
Sloth             VMWLWGISLNAVSLVNLVMXXXXXXXXXXXXXXXXXXXXXGSRVERAEEAL-HMGSSVFS
Hedgehog          VMWLWGISLNAVSLVNLVMSCGISVEFCSHVTRAFTVSTKGSRVQRAEEALAHMGSSVFS
Squirrel          VMWLWGISLNAVSLVNLVMSCGISVEFCSHVTRAFTVSAKGSRVDRAEEALALMGSSVFS


Tenrec            GITLTKFGGIVVLALAKSKIFQIFYFRMYFAMVILGATHGLIFLPVFLSYIGPSINKAKR
Macrobat          GITLTKFGGIVVLAFAKSQIFQIFYFRMYLAMVLLGASHGLIFLPVLLSYIGPSINKAKS
Hyrax             GITLTKFVGIVVLAFAKSQIFQIFYFRMYLAMVLLGVTHGLIFLPVLLSYIGPSINKAKS
Tree shrew        XXXXXXXXXXXXXXXXXXXXXXXXXXXXXXXXXXXXXXXXXXXXXXXXXXXXXPSINKAKS
Armadillo         GITLTKFGGIVVLAFAKSQIFQIFYFRMYLAMVLLGATHGLVFLPVFLSYVGPSVNKARS
Kangaroo rat      XXXXXXXXXXXXXXXXXXXXXXXXXXXXXXXXXXXXXXXXXXXXXXXXXXXXXPSVNKAKR
Shrew             GITLTKFGGIVVLAFAKSQIFQIFYFRMYLAMVLLGATHGLIFLPVLLSYIGPSVNKAKS
Dolphin           GITLTKFGGITVLAFAKSQIFKRFYFRMYFAMVLLGATHGLIFLPVLLSYIGPPINKAKS
Cow               GITLTKFGGIIVLAFAKSQIFQIFYFRMYLAMVLLGATHGLIFLPVLLSYIGPSINKAKS
Pig               GITLTKFGGIVVLAFAKSQIFQIFYFRMYLAIVLLGATHGLIFLPVLLSYIGPSINKAKS
Weasel            GITLTKFGGIVVLAFAKSQIFQIFYFRMYLAMVLLGATHGLIFLPVLLSYIGPSINKAKS
Panda             GITLTKFGGIVVLAFAKSQIFQIFYFRMYLAMVLLGATHGLIFLPVLLSYVGPSINKARS
Dog               GITLTKFGGIVVLAFAKSQIFQVFYFRMYLAMVLLGATHGLIFLPVLLSYIGPSINKAKS
Cat               GITLTKFGGIVVLAFAKSQIFQIFYFRMYLAMVLLGATHGLIFLPVLLSYIGPSINKAKS
Microbat          GITLTKFGGIVVLAFAKSQIFQIFYFRMYLAMVLLGAAHGLVFLPVLLSYIGPSVNKAKS
Horse             GITLTKFGGIVVLAFAKSQIFQIFYFRMYLAMVLLGATHGLIFLPVLLSYIGPSINKAKT
Mouse lemur       GITLTKFGGIVVLAFAKSQIFQIFYFRMYLAMVLLGATHGLVFLPVLLSYIGPSINKAKS
Pika              GITLTKFGGIVVLAFAKSQIFQIFYFRMYLAMVLLGATHGLIFLPVLLSYIGPSTNKAKS
Rabbit            GITLTKFGGIVVLAFAKSQIFQIFYFRMYLAMVLLGATHGLIFLPVLLSYIGPSTNKAKS
Orangutan         GITLTKFGGIVVLAFAKSQIFQIFYFRMYLAMVLLGATHGLIFLPVLLSYIGPSVNKAKS
Human             GITLTKFGGIVVLAFAKSQIFQIFYFRMYLAMVLLGATHGLIFLPVLLSYIGPSVNKAKS
Chimpanzee        GITLTKFGGIVVLAFAKSQIFQIFYFRMYLAMVLLGATHGLIFLPVLLSYIGPSVNKAKS
Gorilla           GITLTKFGGIVVLAFAKSQIFQIFYFRMYLAMVLLGATHGLIFLPVLLSYIGPSVNKAKS
Marmoset          GITLTKFGGIVVLAFAKSQIFQIFYFRMYLAMVLLGATHGLIFLPVLLSYIGPLVNKAKS
Macaque           GITLTKFGGIVVLAFAKSQIFQIFYFRMYLAMVLLGATHGLIFLPVLLSYIGPSVNKAKS
Gibbon            GITLTKFGGIVVLAFAKSQIFQIFYFRMYLAMVLLGATHGLIFLPVLLSYIGPSVNKARS
Baboon            GITLTKFGGIVVLAFAKSQIFQIFYFRMYLAMVLLGATHGLIFLPVLLSYIGPSVNKAKS
Bushbaby          GITLTKFGGIVVLAFAKSQIFQIFYFRMYLAMVLLGATHGLVFLPVLLSYIGPSINKAKS
Tarsier           XXXXXXXXXXVVLAFAKSQINKIFYFRMYLAMVLLGATHGLVFLPVLLSYIGPSINKAKR
Mouse             GITLTKFGGIVVLAFAKSQIFEIFYFRMYLAMVLLGATHGLIFLPVLLSYIGPSVNKAKR
Rat               GITLTKFGGIVVLAFAKSQIFEIFYFRMYLAMVLLGATHGLIFLPVLLSYIGPSVNKAKR
Hamster           GITLTKFGGIVVLAFAKSQIFEIFYFRMYLAIVLLGATHGLIFLPVLLSYVGPSVNKAKR
Guinea pig        GITLTKFGGIAVLAFAKSQIFQIFYFRMYLAMVLLGATHGLIFLPVLLSYIGPSVNKAKI
Opossum           GITLTKFGGIVVLAFARSQIFQIFYFRMYLAMVLLGATHGLIFLPVLLSYIGPSVNKAKS
Tasmanian devil   GITLTKFGGIVVLAFARSQIFQIFYFRMYLAMVLLGATHGLIFLPVLLSYIGPSVNKAKS
Platypus          GITLTKFGGIVVLAFSKSQIFQIFYFRMYLAMVLLGATHGLIFLPVLLSYIGPPVNRAKC
Elephant          GITLTKFGGIVVLAFAKSQIFQIFYFRMYLAMVLLGVTHGLIFLPVLLSYIGPSINKAKS
Wallaby           GITLTKFGGIVVLAFARSQIFQIFYFRMYLAMVLLGATHGLIFLPVLLSYIGPSVNKAKS
Sloth             GITLTKFGGIVVLAFAKSQIFQIFYFRMYLAMVLLGATHGLIFLPVLLSYIGPSINKARS
Hedgehog          GITLTKFGGIVVLAFAKSKIFQIFYFRMYVAMVLLGATHGLIFLPILLSYIGPSINKARS
Squirrel          GITLTKFGGIVVLAFAKSQIFQIFYFRMYLAMVLLGATHGLIFLPVLLSYIGPSLNKAKS

                      1226
Tenrec            STAQERYQDTERERLLNF
Macrobat          LATQERYQGTEREQLLNF
Hyrax             SATQEQYRGTERERLLNF
Tree shrew        RNTQDRYKGTERERLLNF
Armadillo         RTTQERYKGTERERLLNF
Kangaroo rat      HTTQDPYKGTERERLLNF
Shrew             LSTRERYKGTEREHLLNF
Dolphin           LAAQERHRGTERERLLNF
Cow               LTTQQRYRGTEREQLLNF
Pig               LATQERYKGTEREQLLNF
Weasel            LATQERYKGTEREQLLN-
Panda             VATQERYKGTEREQLLNF
Dog               LASQERYKGTEREQLLNF
Cat               LATQEQYKGTEREQLLNF
Microbat          LATQQRHRGTEREQ-LNF
```

Horse	LATQERYKGTEREQLLNF
Mouse lemur	RATEDRYRGTERERLLNF
Pika	CATQERYKGTERERLLNF
Rabbit	CATQERYKGTERERLLNF
Orangutan	CATEERYKGTERERLLNF
Human	CATEERYKGTERERLLNF
Chimpanzee	CATEERYKGTERERLLNF
Gorilla	CATEERYKGTERERLLNF
Marmoset	HATEERFKGTERERLLNF
Macaque	CATEERYKGTERERLLNF
Gibbon	CATEERYKGTERERLLNF
Baboon	CATEERYKGTERERLLNF
Bushbaby	CATEDRYKGTERERLLNF
Tarsier	HATEDRFKGTERERLLNF
Mouse	HTTYERYRGTERERLLNF
Rat	HTTQERYKGTERERLLNF
Hamster	HTTQERYKGTERERLLNF
Guinea pig	HSTRERYRGTERDRLLNF
Opossum	HAAQERNRGTERERLLNF
Tasmanian devil	RAAQERNRGTERERLLNF
Platypus	RAAQERSKGTERERLLYF
Elephant	SATQEQYRGTERERLLNF
Wallaby	HAAQERNRGTERERLLNF
Sloth	RTTQERYKGTEQERLLNF
Hedgehog	LATKERLKGTEREHLLNF
Squirrel	RTTQDRYKGTERERLLNF

Supplemental Figure 2. Branch-site random effects likelihood (branch-site REL) analysis of *NPC1* genes. The hue of each color indicates strength of selection, with primary red corresponding to $\omega > 5$, primary blue to $\omega = 0$ and grey to $\omega=1$. The width of each color component represents the proportion of sites in the corresponding class. Thicker branches have been classified as undergoing episodic diversifying selection by the sequential likelihood ratio test at corrected $p \leq 0.05$

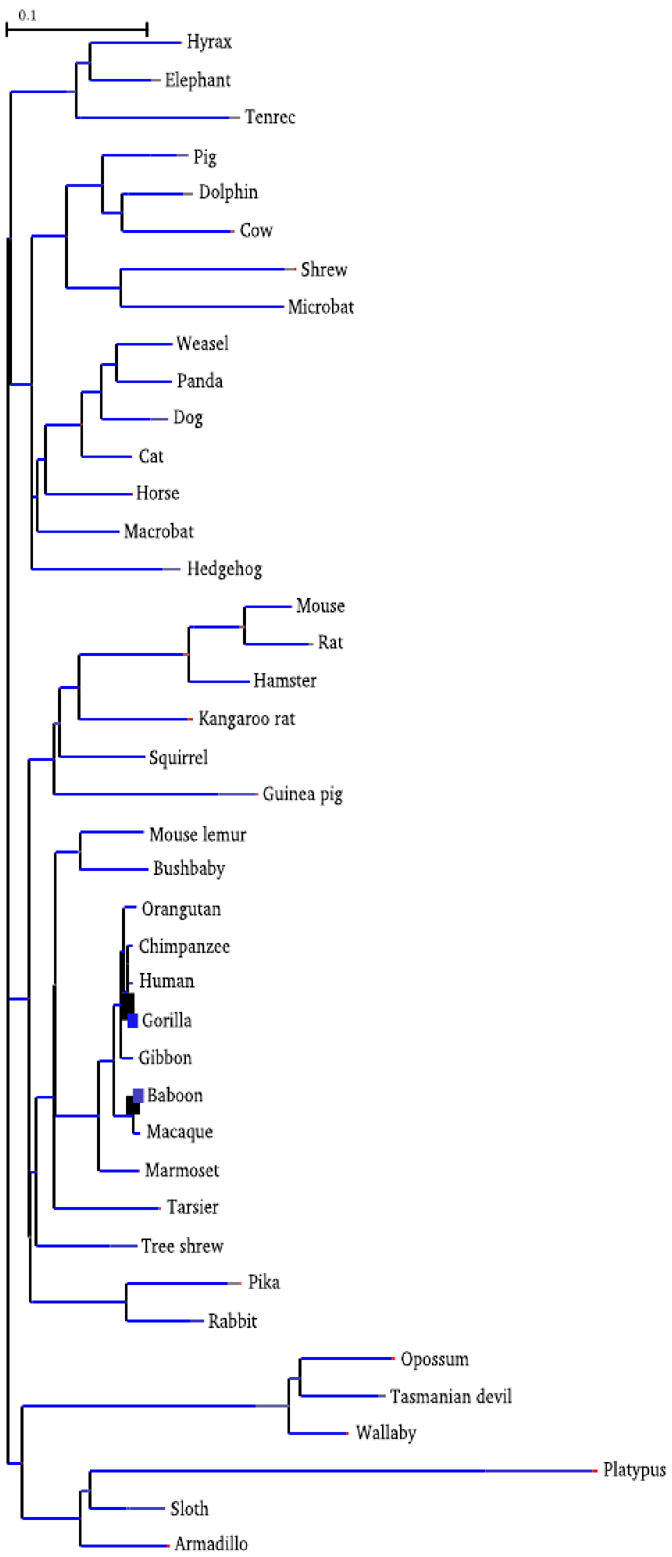

Supplemental Figure 3. Sliding-window analysis of nucleotide diversity along *NPC1* using the 1000 Genomes Project data. θ_W (blue) and π (red) were calculated in sliding windows of 5 kb moving with a step of 500 bp. The 97.5th and the 2.5th percentiles (horizontal dashed lines, blue for $\theta_{W,}$ red of π) were calculated by applying the same procedure to 2000 randomly selected human genes.

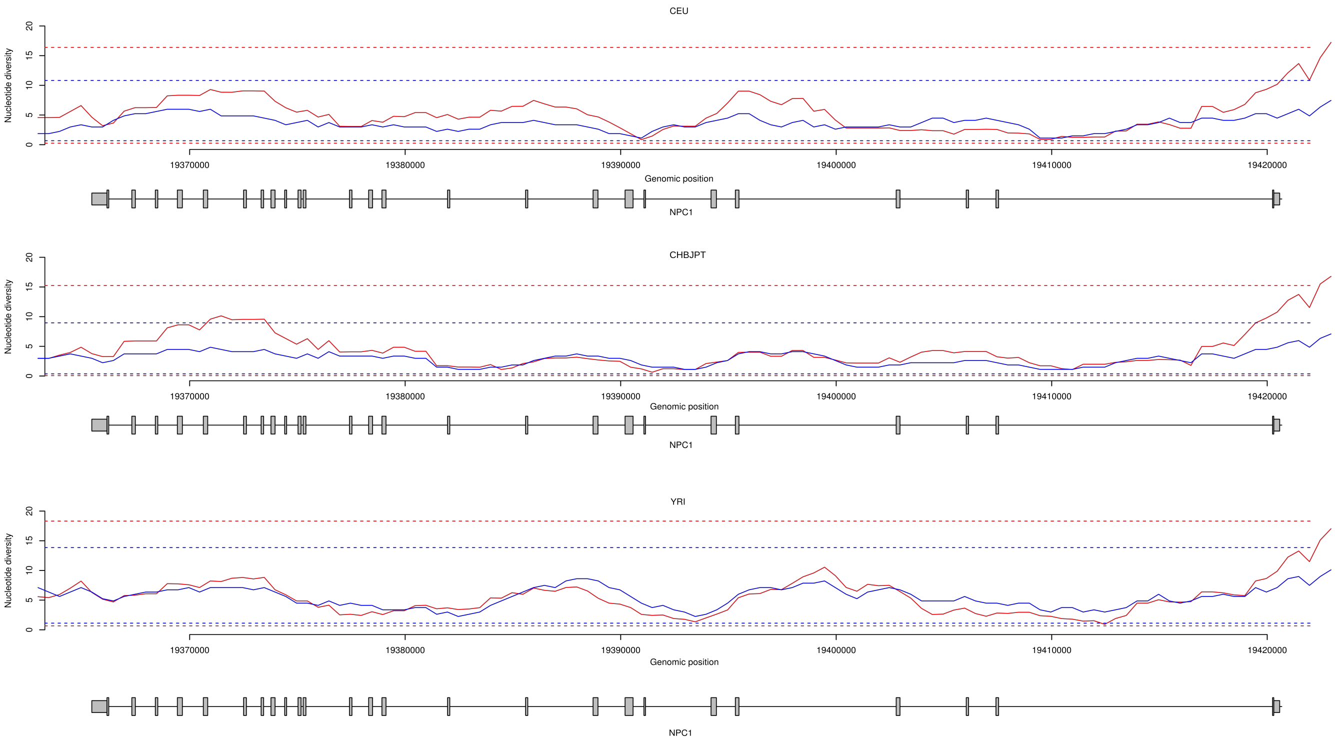

Supplemental Figure 4. Sliding-window analysis of F_{ST} along *NPC1* using the 1000 Genomes Project data. The 1000 Genomes Project data were used to calculate YRI/CEU/CHB-JPT F_{ST} in sliding windows of 5kb moving with a step of 500 bp. Red horizontal lines represent the 97.5th and the 2.5th percentiles in the distribution of F_{ST} calculated for sliding windows deriving from 2000 randomly selected human genes.

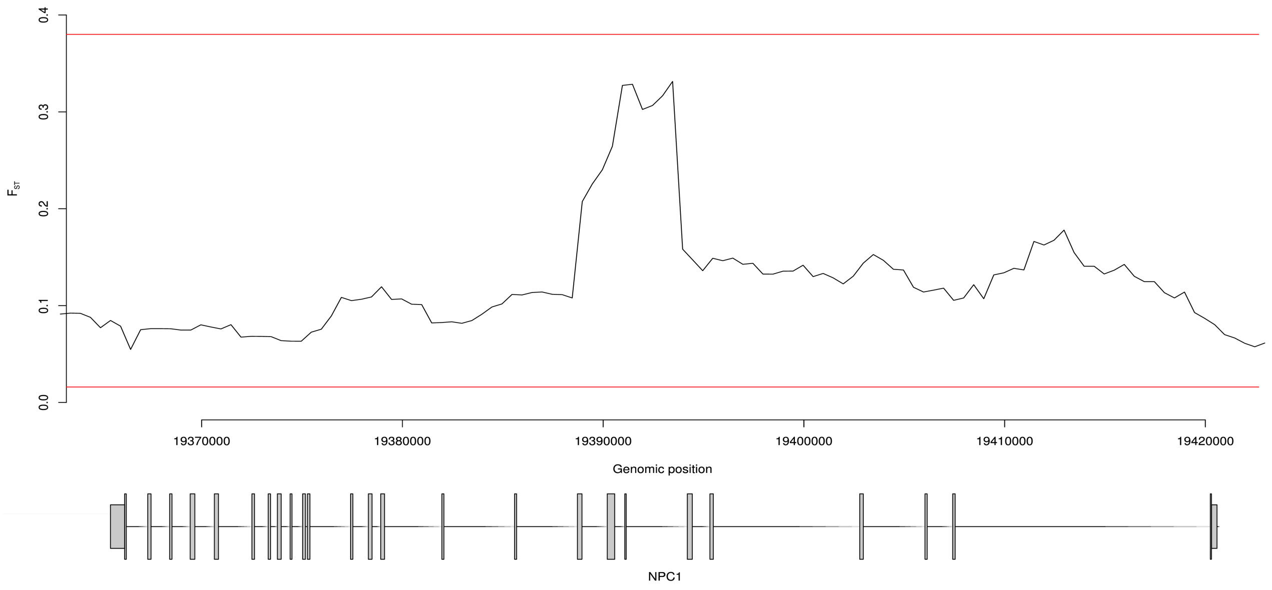

Supplement: Additional file 1 — Table S1: Likelihood ratio test statistics for models of variable selective pressure among sites. The table reports results of the likelihood ratio tests (M7 versus M8 and M8a versus M8) using the F3X4 codon frequency model. Figure S1: Multiple protein alignment of NPC1 mammalian genes. The figure shows the NPC1 multiple species alignment (41 species, Clustal format); positively selected sites and human nonsynonymous polymorphisms are highlighted. Figure S2: Branch-site random effects likelihood (branch-site REL) analysis of NPC1 genes. The figure shows a branch-site REL analysis of NPC1 with the width and color of each branch indicating the strength of selection. Figure S3: Sliding-window analysis of nucleotide diversity along NPC1 using the 1000 Genomes Project data. The figure shows θW and π calculated for Yoruba, Europeans and Asians in sliding windows of 5 kb moving along the NPC1 gene region. Figure S4: Sliding-window analysis of FST along NPC1 using the 1000 Genomes Project data. The figure shows FST (YRI/CEU/CHB-JPT) calculated in 5 kb windows moving along the NPC1 gene region. [file 1741-7015-10-140-S1.PDF]
